# Conserved Water Networks Identification for Drug Design Using Density Clustering Approaches on Positional and Orientational Data

**DOI:** 10.1021/acs.jcim.2c00801

**Published:** 2022-11-09

**Authors:** Jelena Tošović, Domagoj Fijan, Marko Jukič, Urban Bren

**Affiliations:** † Faculty of Chemistry and Chemical Engineering, University of Maribor, Smetanova 17, SI-2000Maribor, Slovenia; ‡ Faculty of Mathematics, Natural Sciences and Information Technologies, University of Primorska, Glagoljaška 8, SI-6000Koper, Slovenia; ⊥ Institute of Environmental Protection and Sensors, Beloruska ulica 7, SI-2000Maribor, Slovenia; # private residence

## Abstract

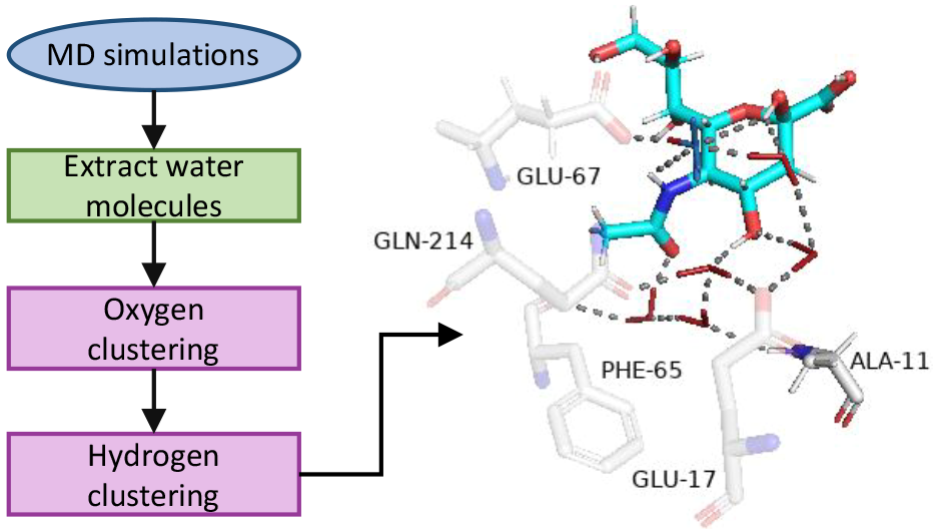

This work describes the development and testing of a method for the identification and classification of conserved water molecules and their networks from molecular dynamics (MD) simulations. The conserved waters in the active sites of proteins influence protein–ligand binding. Recently, several groups have argued that a water network formed from conserved waters can be used to interpret the thermodynamic signature of the binding site. We implemented a novel methodology in which we apply the complex approach to categorize water molecules extracted from the MD simulation trajectories using clustering approaches. The main advantage of our methodology as compared to current state of the art approaches is the inclusion of the information on the orientation of hydrogen atoms to further inform the clustering algorithm and to classify the conserved waters into different subtypes depending on how strongly certain orientations are preferred. This information is vital for assessing the stability of water networks. The newly developed approach is described in detail as well as validated against known results from the scientific literature including comparisons with the experimental data on thermolysin, thrombin, and *Haemophilus influenzae* virulence protein SiaP as well as with the previous computational results on thermolysin. We observed excellent agreement with the literature and were also able to provide additional insights into the orientations of the conserved water molecules, highlighting the key interactions which stabilize them. The source code of our approach, as well as the utility tools used for visualization, are freely available on GitHub.

## 1 Introduction

Nowadays, the important role of water molecules in the ligand-protein binding is widely acknowledged and recognized also in the structure-based drug design. The binding of a ligand to a protein target involves a rearrangement of water molecules in and around the protein binding site.^1^ The first step represents desolvation, i.e. the water shells of both the protein and ligand are partially removed to provide the necessary space for the complex formation. The desolvation process fundamentally affects the thermodynamic profile of binding because it strongly depends on the binding properties of the water molecules before their displacement upon the ligand binding.^2,3^ Besides desolvation, the rearrangement and reordering of water molecules across the protein surface have been intensively studied in recent years.^4^ In particular, the way water molecules rearrange and establish hydrogen bonding networks around the newly formed protein–ligand complex seems to also bear a significant impact on the thermodynamic binding signature.

The influence of the water networks on the thermodynamic signature of the ligand-protein binding was addressed in several previous studies. The interactions between a series of phosphonopeptide inhibitors with the metalloprotease thermolysin were extensively studied and analyzed by high-resolution diffraction experiments and correlated with their thermodynamic binding profiles as measured by the isothermal titration calorimetry.^1,5−10^ The overall conclusion is that there is a direct correlation between the ligand binding affinity and the rearrangement of the water molecules around the investigated inhibitors. Namely, it was observed that the increased stabilization of water molecules through the water networks formation led to the enthalpically more favorable binding signature. A similar effect of the water molecules on the ligand binding in thrombin-2-(aminomethyl)-5-chlorobenzylamide and thrombin-4-amidinobenzylamide complexes was observed by the same authors.^11^ Moreover, Darby et al. explored the contribution of water networks to the ligand binding in the *Haemophilus influenzae* virulence protein SiaP. The authors observed a 1000-fold change in the binding affinity when a single mutation without a direct ligand contact disrupted the nearby water network. They proposed that the perturbation of the water networks can significantly lower the affinity of a ligand-protein binding through the weakening of the enthalpically optimal interactions and the introduction of the solvent mobility.^12^ Studying the mechanism of the enthalpy–entropy (*H*/*S*) compensation in human carbonic anhydrase in complex with a series of benzothiazole sulfonamide ligands with different fluorination patterns, Breiten et al. revealed that differences in the structure and thermodynamic properties of the waters surrounding the bound ligands represented an important contributor to the observed *H*/*S* compensation.^13^

Experimental methods for analyzing the role of water molecules in drug binding face many challenges.^14^ First, the experimental evidence of the intricate solvation effects on the ligand binding is hard to collect. It is also difficult to routinely obtain sufficiently detailed information about water solvation layers from crystal structures.^1^ The assignment of the water molecules in protein diffraction experiments is essential and depends on both resolution and the local ordering phenomena. Yet, another challenge lies in resolving specific water contributions to the binding thermodynamics. Lastly, the water network structures inferred from crystallographic experiments need not reflect a realistic system in an equilibrium solution. On the other hand, computational studies of binding site water molecules represent an alternative approach which can in principle provide a complete, structural, and thermodynamic picture.^14^ Various solvent mapping methods have been developed to detect “important” water molecules and to complement the more rigorous computational approaches (e.g., free energy calculations).^14−17^ Previously developed methods for the detection of important water molecules in active sites can be classified broadly in two groups: static methods (i.e., 3D-RISM,^18^ WATERDOCK,^19^ WaterFLAP,^20^ SZMAP,^21^ and JAWS^15^) and molecular dynamics simulation based methods (ie. ProBiS H2O,^22^ WaterMap,^2,23^ GIST,^24^ AquaMMapS,^25^ WATCLUST,^26^ and WATsite^27−29^).

Only a few studies are devoted to the comparison of the relative success of the water prediction methods. Namely, Bucher et. al compared four solvent mapping methods SZMAP,^21^ WaterFLAP,^20^ 3D-RISM,^18^ and WaterMap^2,23^ by looking at their ability to predict the structure–activity relationships of lead compounds.^17^ Based on the results obtained for three systems (autotaxin and two kinase targets), they concluded that all methods qualitatively reproduced a high energy water molecule near the ligand correctly, with the WaterMap being more accurate in certain cases. In the study of Bortolato et al.,^30^ the water network perturbation in the ligand binding to adenosine A2A antagonists was investigated by means of WaterMap, SZMAP, GRID/CRY probe,^31^ and Grand Canonical Monte Carlo (GCMC) simulations.^16,32−34^ The selected methods were used to predict the position and the relative free energy of the water molecule in the protein active site as well as to analyze the perturbation of the water network as a consequence of the ligand binding. The authors concluded that WaterMap, GRID, SZMAP, and GCMC can be used as complementary tools and that the obtained results can contribute to a better understanding of the structure–activity relationships, both qualitatively (e.g., focusing on water locations) and quantitatively (e.g., predicting relative binding free energies).

Moreover, Betz et al. developed a molecular dynamics (MD) protocol for investigating surface water networks and for predicting solvation sites around different thermolysin-inhibitor complexes.^1,10^ The preferred positions of water molecules were determined using the VOLMAP-plugin in the VMD.^35^ The positions of the oxygen atoms of the water molecules from each time step along the trajectory were binned on a three-dimensional grid. In order to visualize the regions populated above the average by the water molecules during the MD simulation, an average water density map was calculated from the grid. In general, the authors obtained a fairly good correspondence with experimental electron densities from high-resolution crystal structures. Furthermore, the results obtained from the MD simulations containing the initially observed crystallographic water molecules led to slightly better results than those not taking the crystallographic waters into account.

Motivated by the results and the approach of Betz et al.^1^ as well as inspired by the ProBiS H2O method,^22^ we developed a clustering-based python module for the detection of various conserved water types and water networks they form. The methodology involves a comprehensive analysis of the water molecules obtained from MD simulations using a sophisticated clustering algorithm. The unique feature of our method is that it determines the orientations of the hydrogen atoms in addition to the oxygen atom positions. In this way, we can obtain additional information on the water networks. In particular, we can detect which hydrogen bonds are conserved during the MD simulation and consequently form a more stable hydrogen bridge or network. An additional advantage of the developed method is that it does not require the application of the crystal water data to predict water locations but rather relies on the MD simulations only. To test and validate our novel approach, three protein–ligand systems, in which the surface water networks were experimentally observed, were considered, namely: thermolysin, thrombin, and *Haemophilus influenzae* virulence protein SiaP.

## 2 Methods

In this section, the developed workflow is presented. The entire workflow was implemented using the signac framework.^36^ Moreover, the implementation of the methodology used to analyze the conserved water molecules and the classification criteria observed will be thoroughly described. The code for identifying the conserved water molecules from the molecular dynamics simulations is separated into two repositories due to licensing. The hydrogen orientation analysis module can be found at https://github.com/JecaTosovic/ConservedWaterSearch under the BSD3 license, while the module for the visualization and preparation of raw trajectories for this analysis is located at https://github.com/JecaTosovic/WaterNetworkAnalysis under the GPL2 license. Both packages are available on PyPI (via pip).

### 2.1 General Workflow

**Scheme 1:**
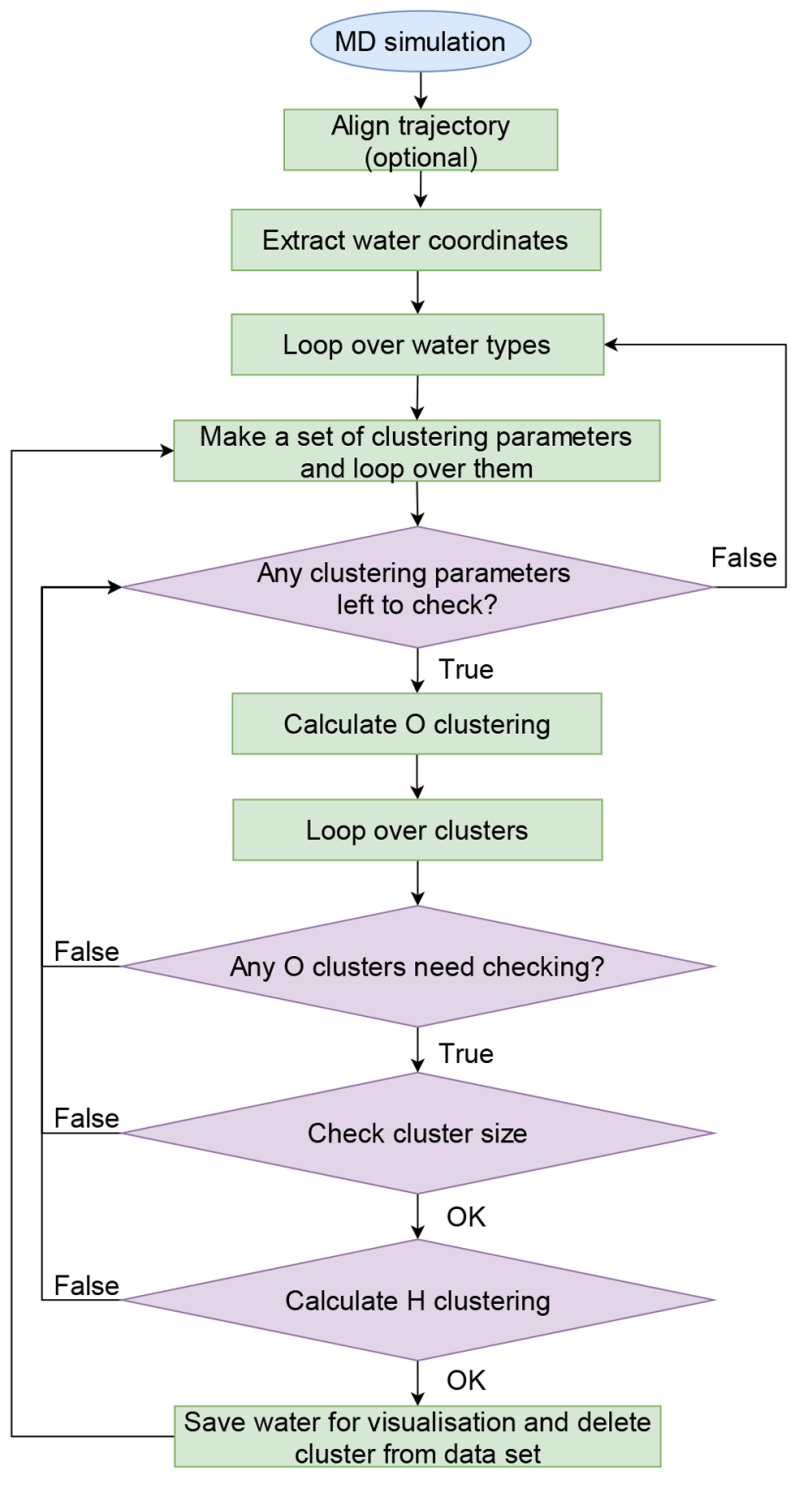
Scheme of a General Workflow of Our Methodology (O and H Denote Oxygen and Hydrogen, Respectively)

The general workflow for identifying key water molecules is presented in Scheme 1. The procedure starts by setting up and running a molecular dynamics (MD) simulation. This is followed by the alignment of the protein structures along the trajectory to a specified frame, if required. Next, in our particular case, the relevant water molecules in a certain radius of a centroid of selected residues (active site amino acids in our case) are extracted. One could, however, select any set of waters from the simulation, such as waters located up to a certain distance from the protein surface or waters at and around an alosteric site, for example. The coordinates of water molecules extracted from the MD snapshots are fed into the oxygen clustering analysis which clusters spatial oxygen data for a given set of clustering parameters using the standard Euclidian *l*^2^ metric. We impose restrictions on the size of clusters chosen for the subsequent analysis to ensure that each cluster actually corresponds to a single conserved water molecule. A strict cutoff in the cluster size such that it cannot be much larger or much smaller than the total number of snapshots in the trajectory is imposed. One would expect that if a water molecule is conserved, it can almost always be found in the same position relative to the protein and the ligand.

**Figure 1:**
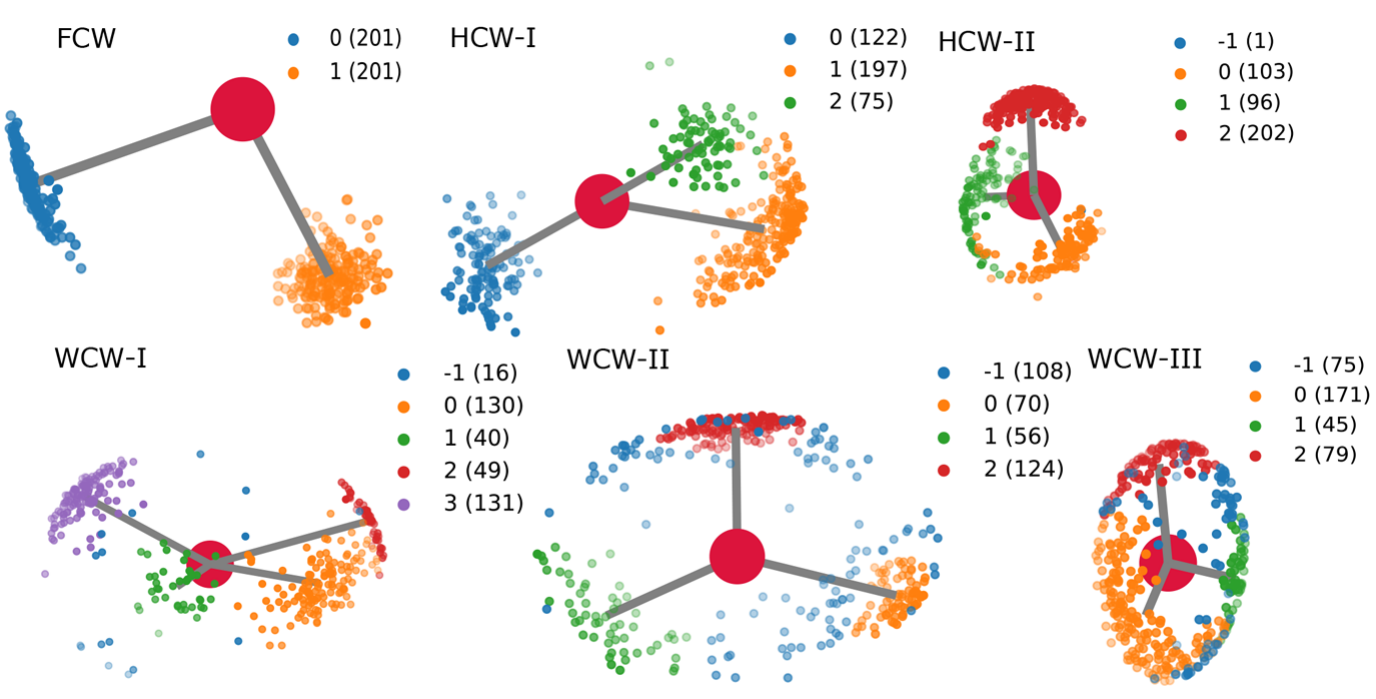
Examples of water types based on hydrogen orientation clustering. The big red sphere represents the oxygen atom. Gray lines represent average cluster orientations. The numbers next to colored points denote the cluster colors and their sizes obtained from the OPTICS clustering of the hydrogen orientations. The −1 cluster contains data points not assigned to any cluster. FCW represents a fully conserved water molecule with two distinct preferred orientations for two identical cluster sizes. HCW-I and HCW-II represent half conserved waters, which show one preferred hydrogen orientation denoted by the largest cluster size as well as several other smaller clusters/orientations which satisfy the water angle constraints. HCW-I represents a scenario in which the second hydrogen switches between the two preferred orientations (two different hydrogen bond acceptors). On the other hand, HCW-II is in the so-called inverse umbrella configuration where one hydrogen has a preferred (main) orientation while the other hydrogen moves in a circle defined by the correct water angle between itself and the main hydrogen orientation. WCW-I, WCW-II, and WCW-III represent weakly conserved water molecules in which 70% of the clusters have to be assigned to valid water orientations, which can be made of different doublets (WCW-I), a triplet (WCW-II), or a circular weakly conserved water (WCW-III).

If an oxygen cluster is of a size similar to the number of snapshots in the trajectory, the hydrogen orientations of the water molecules inside that cluster are analyzed. The clustering of hydrogen orientations of the water molecules is performed to check if the cluster selected in the oxygen clustering procedure belongs to the selected water type or if it is rejected altogether (does not belong to the selected water type). The hydrogen clustering procedure can classify the water molecule into one of three possible types, based on the ensemble of the hydrogen orientations (see Figure 1). The first type is referred to as “fully conserved water” (FCW). In this type, both hydrogen atoms exhibit a unique preferred orientation. The next water type is a so-called “half conserved water” (HCW). In this water type, one hydrogen atom has a unique preferred orientation, whereas the other hydrogen can sample several different orientations. The last water type is a so-called “weakly conserved water” (WCW). There are far fewer orientation restrictions for this water type. Namely, the hydrogen atoms of this water type can have several sets of preferred orientations that again have to adhere to the ideal water angle.

If the hydrogen clustering belongs to a certain water type, a water molecule representing this clustering is saved for subsequent visualization, and either the next oxygen cluster is analyzed or the data belonging to the saved cluster are deleted and oxygen clustering procedure is reset. Then, the results are visualized with PyMOL^37^ or NGLview.^38^ PyMOL represents one of the most popular visualization tools in the biological simulation community, while NGLview is very convenient for the users of IPython/Jupyter notebooks as it can be embedded directly into a notebook.

### 2.2 Molecular Dynamics

All molecular dynamics (MD) simulations were performed in GROMACS,^39^ version 2019.3. The BioExcel Building Blocks (biobb)^40^ were used for setting up and running the selected protein–ligand systems. The biobb software library represents a collection of Python wrappers on top of the popular biomolecular simulation tools.

The following PDB entries were used: 3T73, 3T74, and 3T8G (thermolysin); 2XA5 and 6H76 (SiaP protein); 3RMO and 3RMM (thrombin). To find and add the missing atoms and residues, to remove the unwanted heteroatoms, and to remove the unwanted chains from protein structures, PDBFixer was utilized.^41^ The Amber ff99SB force field was used.^42^ The systems were solvated applying the TIP4P water model.^43^ The pdb2gmx program was used to assign protonation states of amino acid residues.

In order to generate the ligand topology, the missing hydrogen atoms were added using the ReduceAddHydrogens from biobb, which is a wrapper around the reduce function from AmberTools,^44^ and the ligand is minimized using the General Amber Force Field (GAFF),^45^ using the steepest descent from BabelMinimize.^46^ Next, the Python interface to Antechamber^47^—acpype^48^—was applied to generate the ligand force field (GAFF) and topology. In the following step, the new protein–ligand complex which contains both the prepared protein and the ligand was built, and a new combined topology was prepared.

All systems were solvated by explicit water molecules, which were modeled by the TIP4P parameter set, in a cubic box with periodic boundary conditions, and neutralized by adding Na^+^ or Cl^–^. A protein to box distance of 10 Å was used, and the protein was centered in the box. The steepest descent algorithm was used for further energy minimization. Subsequently, the system was heated for 100 ps under constant volume conditions from 100 to 300 K, followed by a 100 ps equilibration under NPT conditions at a pressure of 1 atm and a temperature of 300 K. The Berendsen thermostat^49^ and Parrinello–Rahman barostat^50,51^ were employed. Finally, 20 ns production simulations were performed. The long-range electrostatic interactions were treated using the Particle Mesh Ewald method^52^ with a cutoff of 10 Å, the order of interpolation was 4 with a Fourier spacing of 1.6 Å and a nonbonded cutoff of 10 Å. Atomic coordinates were stored every 10 ps. The LINCS^53^ algorithm was applied to constrain the bonded hydrogen atoms. We opted to study restrained systems in order to be able to compare with the existing results from the scientific literature.^1^ All atoms except water molecules and hydrogens were, therefore, restrained to their initial Cartesian coordinates by a harmonic potential with a force constant of 10 kJ mol^–1^ Å^–2^. The influence of the restraints on the simulation results will be explicitly evaluated in a following study. Although the alignment of the trajectory is not needed for fully restrained systems, our code stack supports alignment either via MDAanalysis^54,55^ or via PROBIS.^56^

### 2.3 Extraction of Water Molecules from Molecular Dynamics Trajectory

Once the trajectory has been aligned (in the case of unrestrained simulations), the relevant water molecules can be extracted for further analysis. We selected all water molecules within a radius of 12 Å around the center of the active site of the investigated protein using MDAnalysis.^54,55^ Additional details on extractions of water molecules are provided in the Supporting Information (see section 1.1 in Supporting Information).

### 2.4 Oxygen Clustering

In this work, we performed oxygen clustering using density based clustering algorithms with the noise OPTICS,^57^ as implemented in the scikit-learn Python module,^58^ although we also support HDBSCAN.^59^ The basics of both algorithms and reasoning on the choice of clustering algorithms are provided in Supporting Information sections 1.2 and 1.3. We have implemented two clustering procedures: a **single clustering procedure** and a **multistage reclustering procedure**. Here, the multistage reclustering procedure will be explained, whereas the details on the single clustering procedure and the problem we encountered applying this approach are provided in Supporting Information section 1.4. The problems encountered when using the single clustering procedure can be circumvented by implementing the multistage reclustering procedure. The multistage reclustering approach can be applied to both OPTICS and HDBSCAN. A set of clustering parameters for oxygen clustering is scanned, until a cluster with hydrogen orientations that belong to one of the water types is found. The obtained water molecule is saved for subsequent visualization. After that, the data belonging to that cluster are deleted from the oxygen clustering data set. The oxygen clustering procedure is reset, and the same set of clustering parameters for the oxygen clustering is scanned again. In this approach, the hydrogen clustering orientation dictates the hierarchy of the selection. The data set is scanned for the water type (hydrogen orientation analysis) with the strictest criteria first, until all waters of this strictest water type are found. Subsequently, the next strictest criteria water type is checked and so on until all water molecules are assigned to a water type or until all clustering parameters and all water types have been exhausted. In this approach, the already used clustering parameters can produce new viable clusters that might be otherwise missed. Deleting the already assigned clusters from the data set improves the further clustering runs immensely because the data that already belong to the selected cluster cannot influence the future clustering runs anymore. The single clustering approach can be thought of as a special case of the multistage reclustering procedure approach where only a single set of clustering parameters is checked. All results presented in this work were obtained with the multistage reclustering procedure approach.

When running the multistage reclustering procedure using OPTICS, the parameter space consists of all possible combinations of a set of ξ = [0.1, 0.05, 0.01, 0.005, 0.001, 0.0005, 0.0001, 0.00001] values as well as a set of minimum sample values, which is in the range of [^1^/_4_*N*_snapshots_, *N*_snapshots_] in increments of 1. The minimum samples parameter represents the number of samples in a neighborhood for a point to be considered as a core point. This parameter also consequently determines the minimum cluster size. ξ defines the minimum steepness on the reachability plot that constitutes a cluster boundary. More details on OPTICS parameters are provided in Supporting Information section 1.2. We note that, as per design, the OPTICS clustering is rather insensitive to the ξ parameter below the default value of 0.05, whereas the minimum samples parameter is a lot more sensitive. We also take advantage of the fact that for a given minimum samples value the clustering calculations for extra ξ values are relatively cheap because the reachability is defined by the minimum samples parameter solely. Thus, we opt to scan both the minimum sample values and ξ in the case of OPTICS.

#### 2.4.1 Oxygen Clustering Selection Criteria

The main condition for selecting the viable oxygen clusters for the subsequent hydrogen orientation clustering analysis is its size. The size is determined by the number of the elements inside the computed cluster. This number is then checked against the number of snapshots used to extract the water coordinates from the MD trajectory. These two numbers should roughly match. A buffer of ±20% on this check is allowed, which ensures that a water molecule is present most of the time in this position.This means that each oxygen cluster is allowed to have *N*_snapshots_ ± 0.2*N*_snapshots_ oxygen atoms. We do this to ensure that the residence time required for a water to be considered conserved is obeyed. Although residence time is not considered directly, the consequence of obeying this conditions has very similar implications on the final result. Because oxygen clustering is performed on a data set consisting of all selected water molecules from all snapshots, in theory, an oxygen cluster can have more oxygen atoms than *N*_snapshots_ by having multiple oxygen atoms contributing to the cluster from the same snapshot. In practice, however, this happens very rarely because of the additional conditions imposed by the subsequent hydrogen orientation clustering. In cases where multiple oxygen atoms from the same snapshots occur, the number of oxygen atoms from the same snapshot is strictly lower than 20% and in practice is usually much lower than 20%. Density based clustering approaches are in general incompatible with the approach where cluster affiliation modifies the availability for the selection of further cluster elements, because this would require the distance matrix to depend on the elements selected for the cluster during the cluster construction process which would result in drastically different clusters depending on which element is selected as the first element of the cluster. This would indeed indicate a water molecule which is a part of a well-defined water network. The position for the oxygen atom of the water molecule is then generated by calculating the centroid of elements in the cluster (defined as the arithmetic mean of the positions of all oxygen atoms in the cluster).

### 2.5 Hydrogen Orientation Analysis

The hydrogen orientation analysis on a given set of orientations can result in four possible outcomes: the hydrogen orientations can belong to a fully conserved water (FCW), a half conserved water (HCW), a weakly conserved water (WCW), or not belong to any conserved water type at all. FCWs show a single special configuration (see Figure 1). HCWs can exhibit several different subtypes, which are apparent from their hydrogen orientations. The first type of HCW can be seen in Figure 1 HCW-I, where one hydrogen is strongly oriented toward one acceptor while the other hydrogen is split between two different acceptors and “jumps” between them. Another type of HCW is the so-called inverse umbrella configuration shown in Figure 1, HCW-II. One hydrogen is again strongly oriented toward a single acceptor while the other hydrogen samples a circle while keeping the optimal water angle. WCWs can on the other hand display a plethora of different configurations similar to the ones already described for the HCW but without a single preferred orientation. The WCW-I in Figure 1 depicts a WCW in which the water jumps between two completely different pairs of orientations (doublets). The WCW-II in Figure 1 shows a similar configuration to that of HCW-I (a triplet), but the orientations are much more scattered. As a consequence, this water is classified as WCW. The last WCW example in Figure 1, WCW-III, represents a so-called “circular” WCW similar to the inverse umbrella HCW-II but without the firm “handle”. The hydrogens in this configuration constantly rotate, swapping between different acceptors while adhering to the ideal water angle.

During the multistage reclustering procedure, we first attempt to identify all waters belonging to a single water type before moving to a different type. The procedure attempts to identify all waters for a given water type until no additional waters of that type can be found. Once all waters of one type are identified, the procedure moves on to the next water type. The order of the water types checked is always FCW, HCW, and WCW. This is because FCW represents a special case of HCW, and HCW again represents a special case of WCW (at least in the technical terms of how they are classified algorithmically). By changing this order, we would lose one type because it would get included in the other type of which it represents a special case. We have also considered the approach where each cluster is consecutively checked for all three water types in a sequence one after another. This approach is viable (and is indeed identical) in the case when a single oxygen clustering procedure is used.

Hydrogen orientation and the classification of water types ensures that the orientations of hydrogen atoms on each selected oxygen clustering conform to one of the three water types described. The following approaches use mainly OPTICS to perform the clustering of hydrogen orientations, with K-means (from scikit-learn^58^) for the FCW analysis. Again, OPTICS was chosen due to its superior clustering quality; however HDBSCAN would have probably worked just as well. Because the data set on which this clustering is performed is much smaller than the clustering set for the oxygen clustering, the speed difference between the two was small enough to select the superior quality clustering algorithm (OPTICS). We have also carried out benchmarking tests to establish the scaling of both OPTICS and HDBSCAN when applied to both the orientation and oxygen position data sets. See Supporting Information section 1.5 for more information.

The hydrogen orientation clustering procedures for determining if a set of water molecules determined from oxygen clustering procedure belongs to one of the three groups outlined earlier is described in detail in Supporting Information section 1.6. The input for analysis of hydrogen orientation are oxygen–hydrogen bond vectors belonging to the waters obtained from the oxygen clustering. The OPTICS clustering parameters are also provided in subsequent sections 1.6.1, 1.6.2, and 1.6.3 of the Supporting Information.

### 2.6 Visualization of Water Molecules

The hydrogen water analysis always returns a set of two hydrogen orientations, the main hydrogen orientation (this is the hydrogen which points in a single direction the whole time if applicable) and a secondary hydrogen orientation. In the cases where many secondary hydrogen orientations can exist (HCW and WCW), a set of hydrogen orientations is returned for each instance of secondary orientations. Each set of water molecules is then constructed from the returned orientation and the oxygen position together with its type are saved for subsequent visualization.

All of the saved water molecules and their types are visualized by constructing a custom PDB file, which contains only the protein structure to which trajectory was aligned, the ligand if present, and water molecules that were identified by the hydrogen orientation analysis. This is performed using MDAnalysis.^54,55^ The main hydrogen (the one with a constant orientation if applicable) is always named “H1” (and translates to a conserved H bond throughout the MD trajectory). Each water’s residue name is changed to one of the water types: FCW, HCW, and WCW, respectively.

This file can then be visualized using NGLview or PyMOL. We visualize the protein as a gray surface; the ligand and the active site amino acids are visualized as licorice (ball and stick in NGLview). If water types are labeled in residue names, FCWs are red, HCWs are blue, and WCWs are green. Additionally, in PyMOL, one can add hydrogen bonds using polar contacts to visualize the hydrogen bond network of the investigated system. Furthermore, we can also optionally visualize the crystal waters from the relevant PDB ID or a water density map for a comparison with results from the scientific literature.

## 3 Results and Discussion

### 3.1 Case Studies

To test and validate our newly developed approach, three protein systems in which the surface water networks were also experimentally observed were studied. In all three cases, our results were compared with the water networks obtained from the crystallographic experiments. For each system, our method also provided additional insight into the mechanism of breakage of the water networks. In addition we compared our results to the MD density water map based approach of Betz et al.^1^

### 3.2 Thermolysin

**Figure 2:**
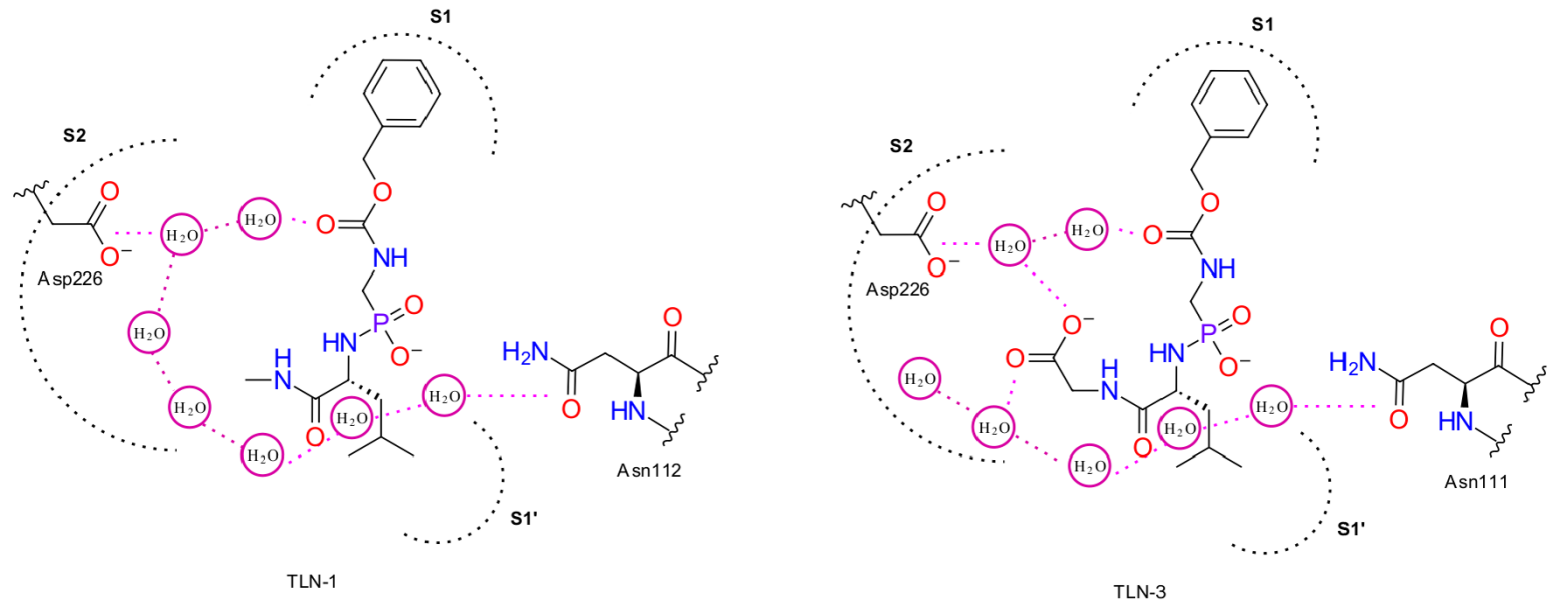
A schematic representation of water networks observed in thermolysin TLN-1 and TLN-3 systems. See below for more details.

In the study of Betz et al., the thermolysin (TNL) protein system in complex with four different inhibitors was investigated.^1^ The inhibitors share a common scaffold, Cbz-Gly-(PO_2_^–^)-l-Leu-NH_2_-P2′ (Cbz = carboxy-benzyl; see Figure S1), but they effectively establish or interrupt a water network depending on terminal substitution (Figure 2).

**Figure 3:**
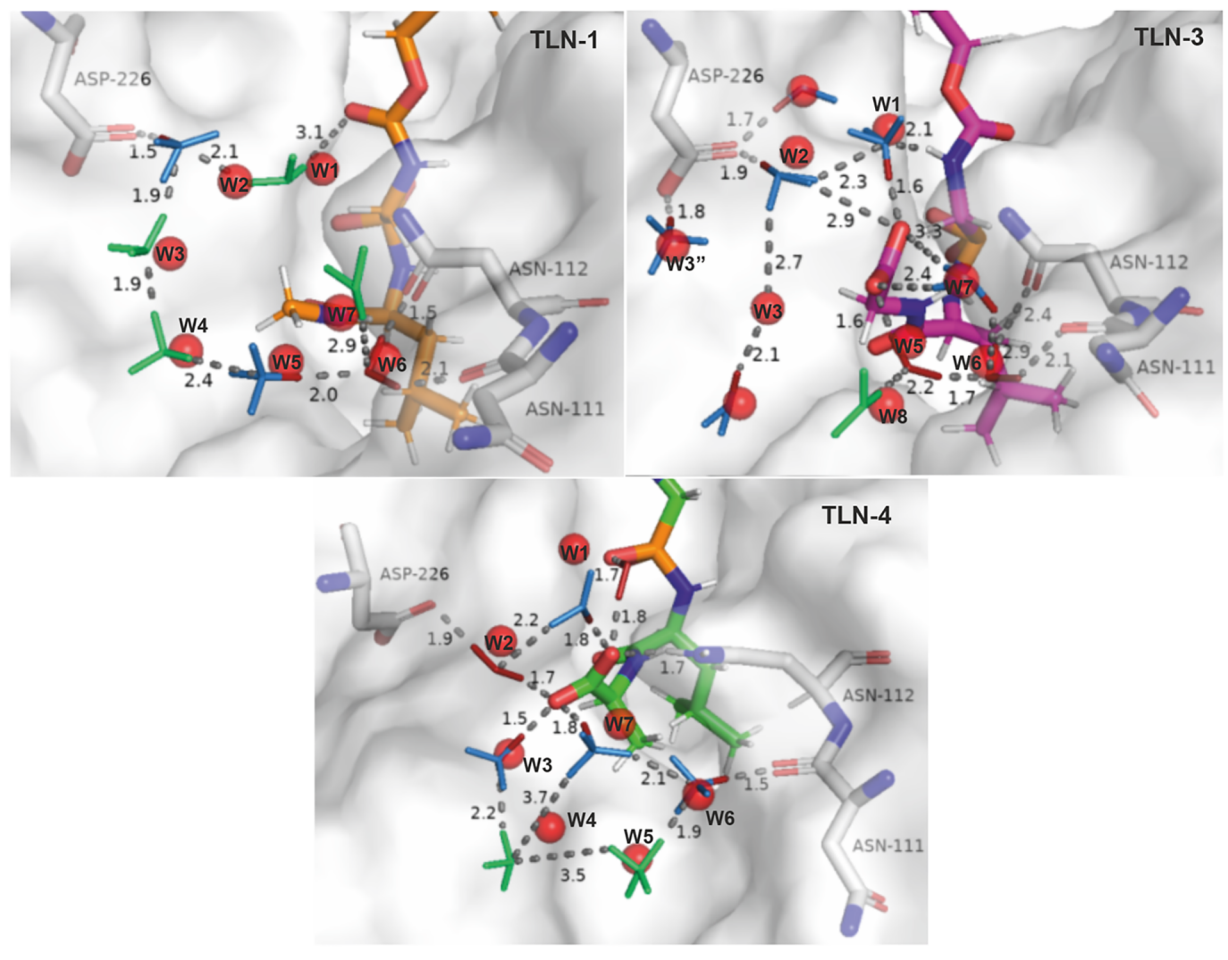
Water networks observed in thermolysin–ligand systems TLN-1,3,4. Red spheres with atom labelling represent the oxygen atoms from the PDB entry obtained from crystallographic experiments. The water molecules which oxygens belong to are involved in the water network according to Betz et al.^1^ for TLN-1. The water molecules from our MD analysis are depicted by small stick models (FCW, red; HCW, blue; WCW, green). The red stick caps denote the preferred (main) orientation in HCW. Important interactions are shown by gray dashed lines with their corresponding hydrogen bond distances reported in Å. Ligands (C, various colors; N, blue; O, red; P, orange; S, yellow; H, white) and key amino acids (C, gray; O, red; N, blue) are depicted as large sticks. The color labeling scheme is the same as in the study of Betz et al.^1^ TLN-1 represents the case in which the ligand (C orange) lacks the methyl and carboxylate groups. A water network was observed in the case of TLN-1 in agreement with the study of Betz et al.^1^ The water network breakage and re-establishment is observed in systems TLN-3 (C magenta) and TLN-4 (C green), respectively.

Therefore, three TLN-ligand systems from the described study were selected, namely, TLN-1 with the extensive water network wrapping around the terminal methyl group, TLN-3, in which a breakage of the water network occurs, and TLN-4 in which re-establishment of the water network was observed (Figure 2). Water positions of all key water molecules involved in the networks of all three studied systems are very well reproduced. In almost all cases, the difference between the crystal and calculated water positions is within 1 Å. The comparison of the water networks calculated using our approach and the experimentally observed water networks is presented in Figure 3.

First, the TLN-1 system, in which the ligand lacks both the methyl and carboxylate groups, is discussed in terms of hydrogen orientation analysis (Figure 3 TLN-1). According to Betz et al,.^1^ the water network in this system is formed from a ligand, W1–W5, (W7)W6, and Asn112. We observe an identical water network with the W2 water position shifted by 1.7 Å toward the Asp226, forming a strong H-bond with its carboxylate (1.6 Å). The W2 water molecule represents an inverse umbrella HCW with the preferred orientation of the H1 atom toward the Asp226. In addition, the W1 water molecule is shifted toward the W2, therefore forming a weak hydrogen bond interaction (3.1 Å) with the carbamate group of the ligand scaffold. The W6 is additionally stabilized with two moderate to strong H bonds with the carbonyl groups of Asn111 (2.1 Å) and Asn112 (2.6 Å). Besides the W2, the W5 water molecule is also characterized as an inverse umbrella HCW type, with the preferred orientation of its H1 atom toward the W6 molecule. We identified most of the water types in this system as WCW and HCW, with the exception of W6, which is of FCW type, indicating a weak water network connected via weak hydrogen bonds.

In the case of TLN-3 and TLN-4 with terminal carboxylate (deprotonated at physiological pH as per Betz et al.^1^ and confirmed by Jaguar (Schrödinger SMD, LLC, New York, USA)^60−62^ calculation of p*K*_a_ = 4.86; using self-consistent field (SCF) methodology), the disruption of the water network in the TLN-3 system occurs when a carboxylate at the R2 position is introduced (Figure 3, TLN-3). Namely, the W4 water molecule is displaced and the gap between the W3 and W5/W8 water molecules is formed. Moreover, the W3 water molecule is shifted to facilitate the contact with the introduced carboxylate. In TLN-3 (Figure 3 TLN-3 top right), we observe the same water network breakage as in Betz et al.^1^ We again elaborate further on hydrogen bond orientations and strengths based on our hydrogen orientation clustering. The W1 water which is of HCW type has its main hydrogen oriented toward the carboxylate group forming a strong hydrogen bond (1.6 Å). The W2 molecule is also identified as HCW water with main hydrogen oriented toward the carboxylate oxygen of Asp226 (1.9 Å). Our approach does not classify W3 water as a conserved water. In order to further understand this discrepancy, we conducted a water density map analysis and found that there should be a water present at this location (see Figure S4). Next, we tested our clustering approach in “oxygen” only clustering mode and confirmed that the clustering scheme indeed detects a valid cluster of oxygen atoms at the position of W3 (see Figure S4A). However, when we further analyzed the hydrogen orientations of this oxygen cluster, we found that hydrogen orientations were too spread out for the oxygen cluster to be considered FCW, and that cluster sizes and their relative angles did not satisfy HCW or WCW (see Figure S4B). This suggests that the W3 water often reorients itself, causing constant breakage of the water network at this point in space. The W5 water is detected as FCW water with strong hydrogen bonds toward the carboxylate (1.6 Å) and toward W6 (1.7 Å). The W6 water is also identified as FCW with its hydrogens oriented toward C=O groups in Asn111 and Asn112. The W7 capping water is also detected as HCW with it is main hydrogen oriented toward W6, while the other hydrogen’s orientation is swapping between the two oxygens in the ligand’s carboxylate. Finally, W8 is also detected as WCW. In conclusion, the water network detected in our simulations matches the one from Betz et. al, and the breakage in the water network between W3 and W5 and W8 is also reproduced. The results are also corroborated by a comparison of water density maps from our simulations to those of Betz et al. where excellent agreement can be observed (see Figure S3).

In the case of TLN-4, we also maintain good agreement with results of Betz et al. The main difference is in the W1, which is detected as two waters in case of our simulations—a FCW and HCW. The FCW shows two strong hydrogen bonds forming a bridge between the carboxylate group and phosphate group, one toward the carboxylate oxygen of the ligand (1.8 Å) and one toward the oxygen of the phosphate group of the ligand (1.7 Å). The HCW’s main hydrogen (the one with preferred orientation) is also oriented toward the same carboxyl oxygen of the ligand. The W2 is detected as FCW, which bridges carboxylate oxygens of the ligand and Asp226, respectively. The W3 water molecule is of the inverse umbrella HCW type with the preferred orientation of the main H atom toward the ligand’s carboxylate oxygen. The W3 molecule is connected with W4 of the WCW type with a moderate H bond. Our clustering analysis predicted a shifted W4 (WCW) water position in comparison to the crystal water molecule. As can be seen from Figure S5, an elongated water density profile which corresponds to two different crystallographic waters (labeled W4 and W4′ in Figure S5) was detected as a single water molecule in our clustering approach, signaling that crystallographic waters W4 and W4′ might be a single water molecule dislocated between these two water sites. The W4 forms weak hydrogen bond with W5 of the WCW type (3.5 Å), which is further connected with an inverse umbrella HCW (W6) through a strong H bond (1.9 Å). The preferred orientation of the main hydrogen of W6 is toward Asn111 (1.5 Å). The W6 forms a moderate hydrogen bond (2.1 Å) with inverse umbrella W7 of the HCW type. The main hydrogen is oriented toward ligand’s carboxylate, forming a strong hydrogen bond (1.8 Å).

A key takeaway obtained from our method is the transient nature of the observed water network. Namely, the detailed analysis of the individual water conservation produces waters of the FCW, HCW, and even WCW types, and we postulate that full dynamic evaluations of similar systems should be done in the future to further study the nature and dynamics of such networks and their influence on ligand affinity. To provide additional validation of our method, we have compared results presented here with the water density map approach from the work of Betz et al. in Supporting Information section 2.2.1.

### 3.3 Thrombin

**Figure 4:**
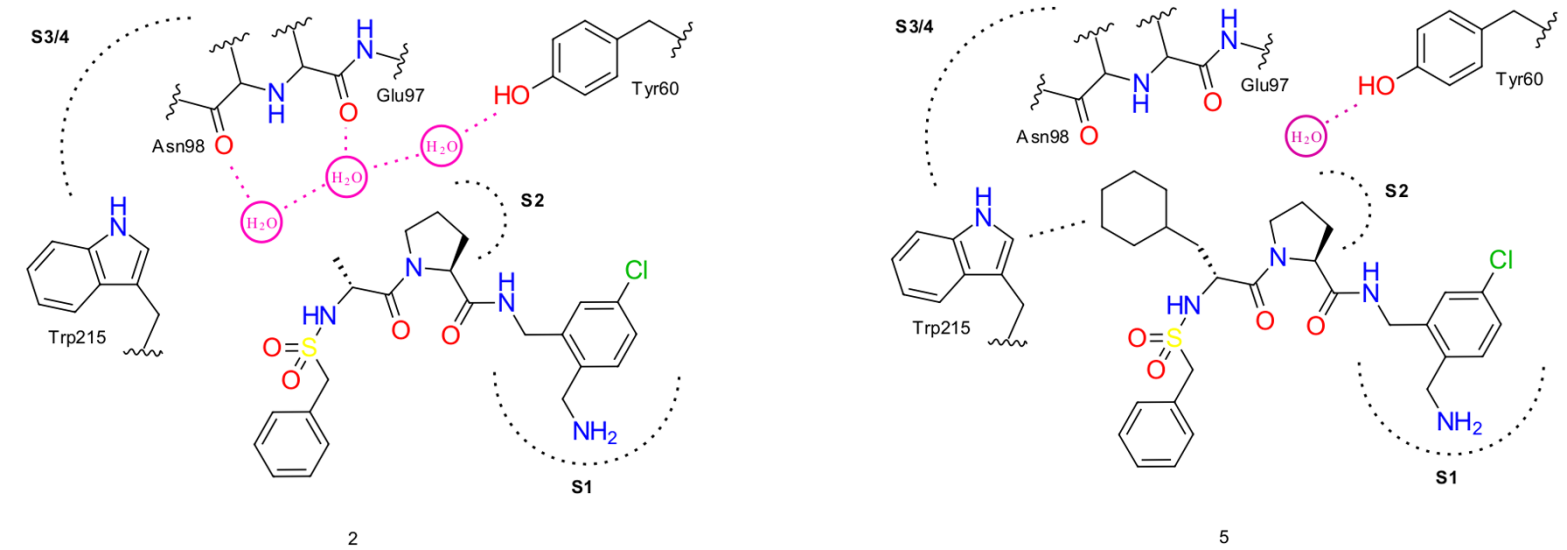
A schematic representation of water networks observed in thrombin-ACB 2 (R: methyl) and 5 (R: cyclohexylmethyl) systems. See below for more details.

To study the role of water in ligand binding, Biela et al. investigated the hydrophobic S3/4 pocket of thrombin.^11^ For this purpose, a series of the thrombin-ACB (2-(aminomethyl)-5-chlorobenzylamide) complexes were synthesized (Figure S2). The P3 substituent was systematically varied by hydrophobic residues (Gly, d-Ala, d-Val, d-Leu, and (S)-2-amino-3-cyclohexylpropanoic acid). In the cases of Gly, d-Ala, d-Val, and d-Leu as P3 substituents, a similar water network is preserved. This is, however, disrupted when a cyclohexylmethyl moiety is introduced as the P3 substituent. Thus, we additinally explored the systems with methyl and cyclohexylmethyl substituents (Figure 4).

**Figure 5:**
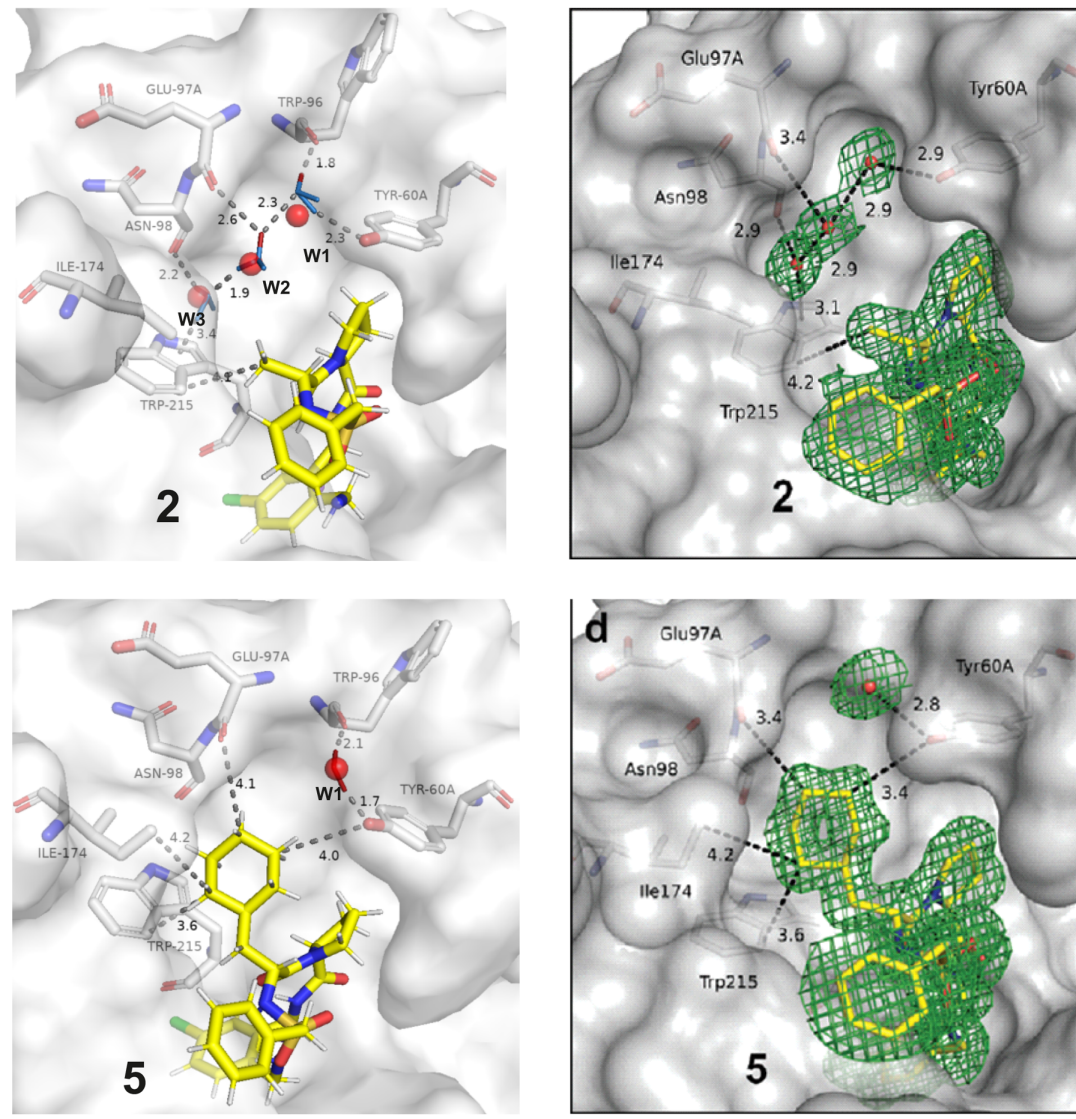
Comparison of water networks observed in thrombin–ligand systems in our study (left) and the experimental study of Biela et al.^11^ (right). Red spheres with atom labeling schemes represent the oxygen atoms from the PDB entry which correspond to the crystallographic experiments reported by Biela et al.^11^ Water molecules from our analysis are shown in small stick models (FCW, red; HCW, blue). Red stick cap depicts the preferred (main) orientation in HCW. Important hydrogen bond and van der Waals interactions are denoted by dashed lines with their corresponding lengths reported in Å. Ligands (C, yellow; N, blue; O, red; Cl, green) as well as the key amino acids (C, gray; O, red; N, blue) are shown as large sticks. The systems (**2** and **5**) and the color labeling scheme are the same as in the study of Biela et al.^11^**2** represents the case in which a ligand has a d-Ala substituent as P3, while **5** represents the ligand with a cyclohexylmethyl substituent as P3. We observed a well-defined water network in the case of **2** and a broken water network in the case of **5**.

First, the water network in the thrombin-ACB with d-Ala substituent system is described (Figure 5 (**2**)). The water network consists of three water molecules of the HCW type that are further connected to the amino acid residues of the active site. The first water molecule W1 is located close to the rim of the S3/4 pocket. The preferred orientation of the main hydrogen in this water molecule is directed toward the Trp96 carbonyl group with which it forms a strong hydrogen bond (1.8 Å). The other hydrogen orientation of W1 is directed toward Tyr60A as reported by Biela et al, but in our case it is split into two distinct hydrogen orientation clusters. The neighboring water molecule W2 is positioned between W1 (H-bond distance = 2.3 Å) and W3 (H-bond distance = 1.9 Å). It is also connected with the carbonyl group of Glu97A (H-bond distance = 2.6 Å). The third water molecule W3 is bound to the backbone carbonyl of Asn98 (H-bond distance = 2.2 Å) and forms an additional polar π interaction with the Trp215 indole moiety (3.4 Å).

When the cyclohexylmethyl substituent is introduced (Figure 5 (**5**)), significant alterations in the water network occur. In this case, the steric interference of the cyclohexyl ring causes the displacement of two water molecules and consequently induces a breakage of the network. Only the W1 molecule located at the boundary of the pocket remains. From the hydrogen orientation analysis, we can provide some additional details about the key interactions in the studied systems. Namely, the displacement of W2 (and W3) molecules and the breakage of the water network induces the change in the W1 molecule type from HCW to FCW and a key Trp96 H-bond with W1 missed in the original experimental work.^11^ Furthermore, the conformation of the cyclohexyl ring is stabilized by several van der Waals interactions: Trp215 (3.6 Å), Ile174 (4.2 Å), Glu97A (4.1 Å), and Tyr60A (4.0 Å). The obtained results are thus in a perfect agreement with the observed water networks in the experimental study of Biela et al. where individual water conservation and interaction patterns are now also elaborated.^11^ Finally, we also compared our clustering result approach to the water density map approach (see Supporting Information 2.2.1, Figure S6) and obtained excellent agreement.

### 3.4 *Haemophilus influenzae* Virulence Protein SiaP

**Figure 6:**
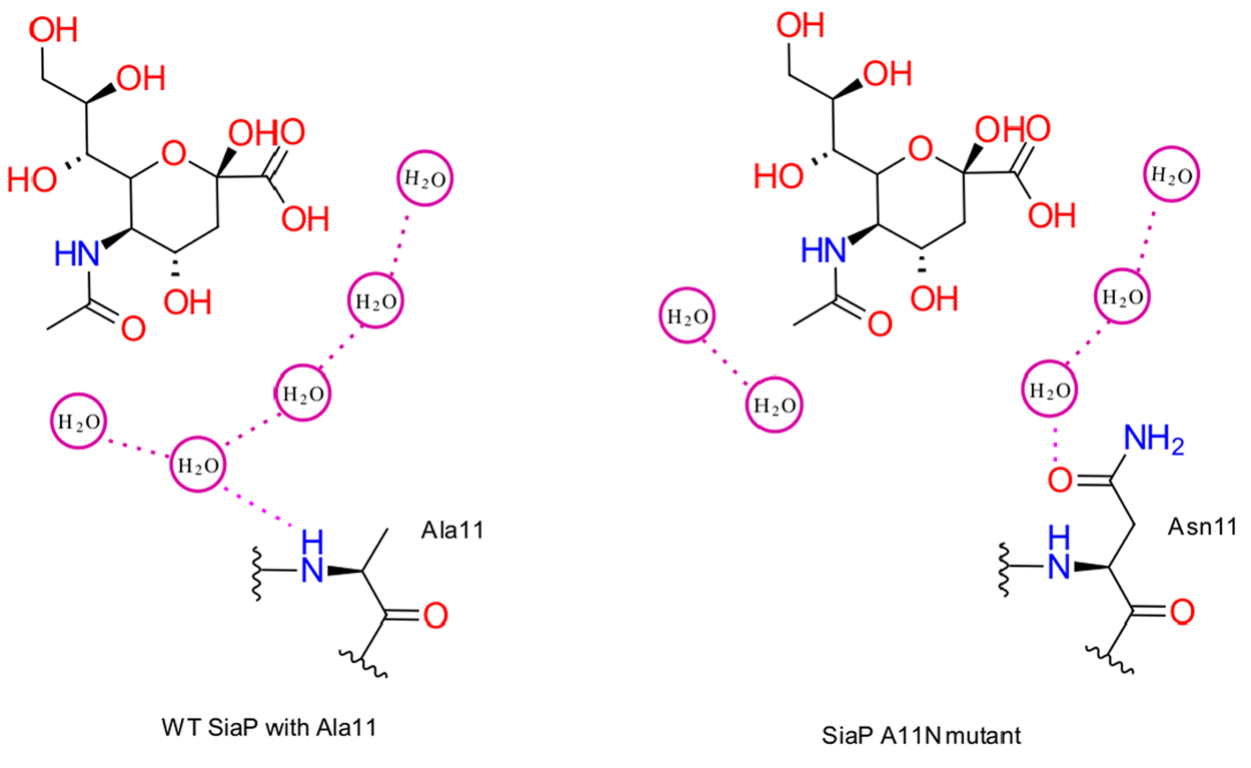
A schematic representation of water networks observed in the wild type SiaP (Ala11) and in the SiaP mutant (A11N) systems. See text for more details.

**Figure 7:**
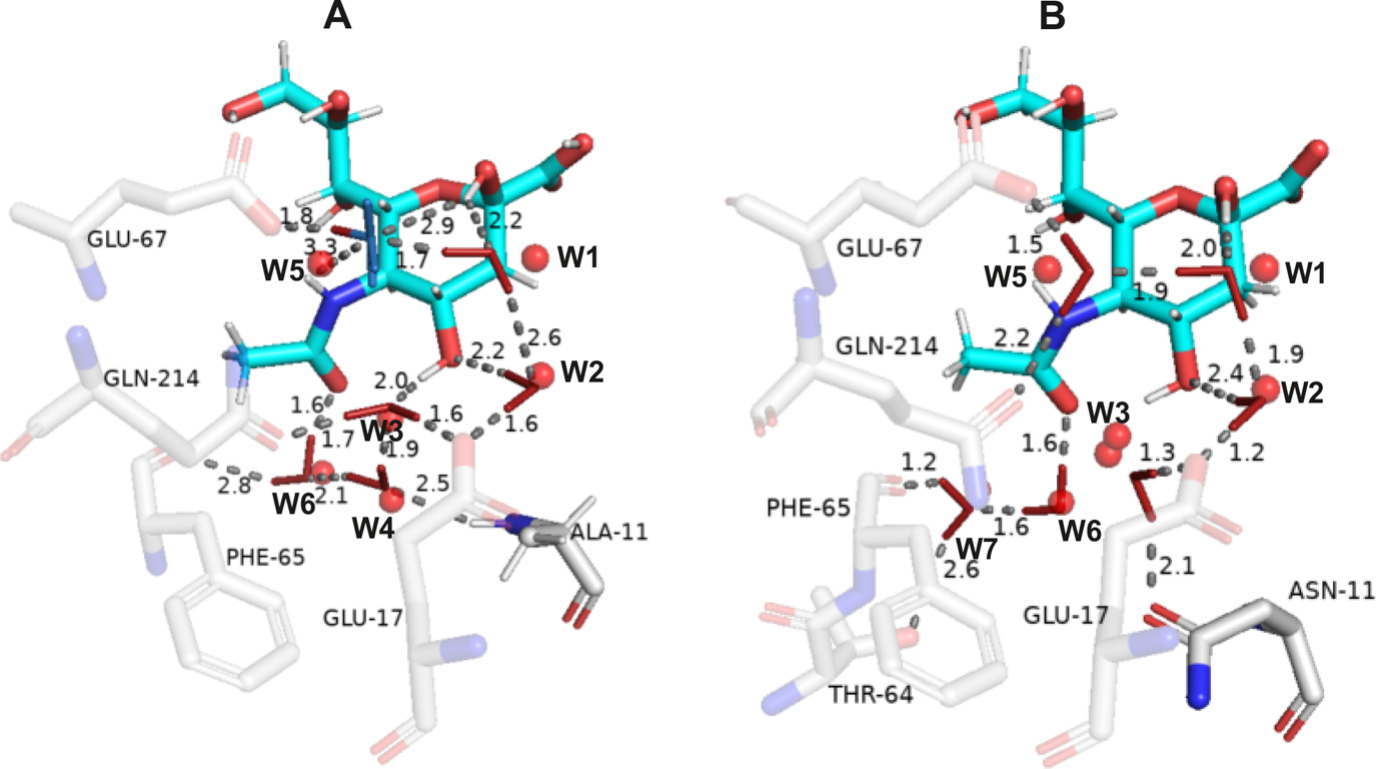
Water networks observed in SiaP (A) WT and (B) A11N mutant bound to Neu5A. Red spheres with atom labeling schemes represent the oxygen atoms from the PDB entry which correspond to the crystallographic experiments reported by Darby et al.^12^ Water molecules from our analysis are depicted by a small stick model (FCW, red; HCW, blue). Red stick caps denote the preferred (main) orientation in HCW. Important hydrogen bond interactions are displayed by dashed lines with their corresponding lengths reported in Å. Ligands (C, cyan; N, blue; O, red) as well as the key amino acids (C, gray; O, red; N, blue) are shown as large sticks. The water labeling scheme is the same as in the experimental study of Darby et al.^12^ We observed a well-defined water network in the case of A, while B shows a broken water network, all in perfect agreement with the study of Darby et al.^12^ Additionally, we identified all of the key interactions from hydrogen orientation analysis between conserved waters and the key amino acids in the active site. See the main text for more details.

In the study of Darby et al., the differences in the water network in system where the sialic acid (N-acetylneuraminic acid; Neu5A or NANA) binds to the wild-type SiaP (SiaP WT) and to the SiaP A11N mutant was studied (Figure 6) .^12^ Namely, upon the binding of Neu5a, the SiaP protein undergoes a major conformational closure to entrap 10 water molecules. In the case of the WT, the water network consisting of five water molecules was observed (Figure 7A). However, Asn11 of the mutant SiaP disrupts the water network observed in the WT with the relocation of the W4 and the rearrangement of the waters W3 and W6 (Figure 7B). The rupture of the water network due to the Asn11 mutation caused Neu5A *K*_d_ to rise from 30 nM to 42 μM, a difference of more than 3 orders of magnitude (consisting of Δ*H* of 12.4 kcal mol^–1^ and – *T*Δ*S* of −8.0 kcal mol^–1^ at 298 K).

Our methodology demonstrates that the SiaP water network consists of the water molecules of the fully conserved type (FCW) with the only exception being the half conserved water (HCW) molecule W5 (Darby et al. numbering) in the WT SiaP. All water molecules in both systems are thus connected through strong to medium hydrogen bonds with well-defined orientations. Our FCW, HCW, and WCW water distinction clearly defines the orientation behavior of individual waters and thus offers insights into the persistence of the formed water networks during the simulation time period. W1 and W2 water molecules thus remain unperturbed when mutating Ala11 to Asn. W2, W3, and W6 are additionally stabilized by strong H bonds with the amino acid side chains. We report that, besides the absence of the W4 molecule in the water network observed in the SiaP A11N mutant, the largest difference in comparison to the WT is in the different orientation of the W3 (FCW) molecule. Due to the displacement of the W4, the water network is disrupted at the hydrogen bond position between the W3 and W4 molecules, which in turn influences the W3 orientation in the A11N mutant. In contrast to the WT case in which the W3 is oriented toward Gln214 and Glu17, in the A11N mutant, the W3 forms a strong hydrogen bond with the carbonyl group of Asn11 not present in the WT. The W5 molecule also changes to the FCW type and forms H bonds with W1 on the one side and with Gln214 and Glu67 on the other side, forming a W4-missing network analogous to the WT.

As can be seen from Figure 7, the experimentally observed water network and the water network obtained in the present study are in perfect agreement. We can further elaborate on a complete remodelling of the water network and postulate a detailed analysis of the water network topolgy via individual water types pinpointing a detailed orientation analysis as presented herein. We can thus substantiate the mechanism of the formation and breakage of the observed water network in the time domain and its utmost importance for the Neu5A binding affinity. The authors note that similar phenomena can provide a detailed understanding of protein–ligand contacts in the future. Last but not least, we also compared our clustering approach results to the water density map approach (see Supporting Information 2.2.1 Figure S7) and obtained excellent agreement.

## 4 Conclusions

In the present study, we present a methodology for classifying and detecting conserved surface water networks in protein–ligand complexes. The general workflow of our novel approach is as follows. First, the investigated protein–ligand system in water is simulated using molecular dynamics. After the trajectory is aligned, water coordinates in observed regions of the simulated system are extracted. In the next key step, we perform a newly developed multistage reclustering procedure on water positional data. In the last step, a novel analysis of hydrogen orientations is performed where groups are classified according to the preference of hydrogen orientations toward the receptor. This protocol allows a strict conserved water identification and classification into three groups, namely, a fully conserved water (FCW), a half conserved water (HCW), and a weakly conserved water (WCW), where roles and contributions of individual waters to the formed water network can be studied.

Our methodology was tested against three different systems: thermolysin,^1^ thrombin,^11^ and *Haemophilus infuenzae* virulence protein SiaP,^12^ where the surface water network as well as its disruption were also observed experimentally. An excellent agreement between our computations and the experimentally obtained results was achieved giving us confidence in the validity of the devised approach. Moreover, we are able to provide additional information on the specific key interactions which stabilize the water network due to our multistage clustering and hydrogen orientation analysis. This can prove useful when designing and improving the ligand binding by substantiating the choice for ligand elaboration in a way that complements the organization of the water networks.

The main advantage of our methodology as compared to already existing methods for identification of important water molecules is its ability to extract preferred and possible orientations of hydrogen atoms in addition to the positions of oxygen atoms. Another benefit is the proposed classification of conserved water types, which can have big implications on the stability of the water network. The prevalence of weakly conserved waters (WCW) with very loose orientations in the water network signals that the stability of such a network is probably lower than ones made of half (HCW) and fully conserved (FCW) waters. Although we chose to study protein–ligand systems in this work, the methodology presented here is quite general and could be applied to study water networks at any types of surfaces. Likewise, since the method successfully identifies conserved waters and their networks, it could also serve for the hotspot or binding site identification, and this will be elaborated upon in our further research. The influence of conformational changes in the ligand or active site amino acids on the water network will be explored further in a subsequent study as well. Although our methodology does not calculate free energy contributions of water molecules to the binding, it could serve as a starting point and data source for the development of better free energy calculation methods.

## Notes

Data and software are available free of charge. The hydrogen orientation analysis module can be found at https://github.com/JecaTosovic/ConservedWaterSearch under the BSD3 license, while the module for the visualization and preparation of raw trajectories for this analysis is located at https://github.com/JecaTosovic/WaterNetworkAnalysis under the GPL2 license. The examples and documentation are available on https://waternetworkanalysis.readthedocs.io/en/latest/ and https://conservedwatersearch.readthedocs.io/en/latest/. Both packages are available on PyPI for convenience. The signac python scripts used to obtain the results can also be found attached as Supporting Information files.

## Supporting Information Available

Signac python scripts (ZIP)
Details on extraction of water molecules from MD trajectories; short overview of clustering algorithms; reasoning of the choice of clustering algorithms; single clustering procedure; benchmarking of clustering algorithms; detailed explanation of hydrogen orientation analysis; structural formula of thermolysin ligand Cbz-Gly-(PO_2_^–^)-l-Leu-NH_2_-P2′; structural formula of thrombin ligand ABC (2-(aminomethyl)-5-chlorobenzylamide); calculation of water probability density maps from our simulation results; comparison of probability density maps calculated from our trajectories with calculated water probability density maps using simulations from the scientific literature (PDF)

## Abbreviations

MD: molecular dynamics
3D-RISM: 3D reference interaction site model
SZMAPS: solvent-zap-mapping
JAWS: just add water molecules
ProBiS: protein structure binding sites
GIST: grid inhomogeneous solvation theory
GCMC: grid canonical Monte Carlo
GAFF: general Amber force field
TIP4P: transferable intermolecular potential 4P
PME: particle mesh Ewald
LINCS: linear constraint solver for molecular simulations
OPTICS: ordering points to identify the clustering structure
HDBSCAN: hierarchical density-based spatial clustering of applications with noise
FCW: fully conserved waters
HCW: half-conserved waters
WCW: weakly conserved waters
TLN: thermolysin
ACB: 2-(aminomethyl)-5-chlorobenzylamide
SiaP: *Haemophilus influenzae virulence* protein SiaP
WT: wild type
