# Supplementary material for: Conserved Water Networks Identification for Drug Design Using Density Clustering Approaches on Positional and Orientational Data
Source: J Chem Inf Model. 2022 Nov 9;62(23):6105–17. doi: 10.1021/acs.jcim.2c00801 (PMC9749026; doi:10.1021/acs.jcim.2c00801)
Supplement: Supplementary file 2 — ci2c00801_si_002.pdf [file ci2c00801_si_002.pdf]

# Supporting Information: Conserved Water Networks Identification for Drug Design Using Density Clustering Approaches on Positional and Orientational Data

Jelena Tošović,[†,§] Domagoj Fijan,[‡,§] Marko Jukič,[*,†,¶] and Urban Bren[*,†,¶]

†*Faculty of Chemistry and Chemical Engineering, University of Maribor, Smetanova 17, SI-2000 Maribor, Slovenia*

‡*Current address: Department of Chemical Engineering, University of Michigan, Ann Arbor, Michigan 48109, United States*

¶*Faculty of Mathematics, Natural Sciences and Information Technologies, University of Primorska, Glagoljaška 8, SI-6000 Koper, Slovenia*

§*These authors have contributed equally.*

E-mail: marko.jukic@um.si; urban.bren@um.si

Phone: : +386 2 2294 428 ; : +386 2 22 94 421

## 1 Details on newly proposed methodology

### 1.1 Details on extraction of water molecules from MD trajectories

Prior to the extraction of the water molecules, the simulation box was centred in such a way that all the selected water molecules are inside the same periodic image. This is important because the periodic boundary conditions are not imposed when calculating the $l^2$ distance

between the points in the subsequent oxygen clustering analysis. The centre of the active site can be determined in multiple ways. One could calculate the centroid of Cartesian coordinates of the ligand bound to the protein. This is, however, impractical for studies without a ligand. Thus we opted to apply a set of amino acids which define the active site and calculates their centroid to determine the centre of the active site.

## 1.2 Short overview of clustering algorithms used in this study

The parameter space for OPTICS consists of a minimum samples parameter and $\xi$ parameter. DBSCAN uses $\epsilon$ instead of $\xi$ and also has minimum samples parameter. Minimum samples parameter represents the number of samples in a neighbourhood for a point to be considered as a core point. This parameter also determines the minimum cluster size consequently. $\xi$ on the other hand determines the minimum steepness on the reachability plot that constitutes a cluster boundary. In OPTICS/DBSCAN "language" the reachability is the core concept used to determine the affiliation of a point to a cluster. The reachability graph is simply a graph of distances (reachabilities) between each successive pair of data points as processed by the OPTICS algorithm. From this graph a DBSCAN result can be extracted by imposing a strict cutoff for reachability (this is $\epsilon$ in DBSCAN "language") cutting the graph in different clusters. In OPTICS instead of a strict cutoff, $\xi$ parameter is used, which cuts the reachability graph depending on steepness of the reachability allowing for variable cluster densities.

Core distance is another key quantity used in OPTICS and HDBSCAN "language". It is determined by the nearest distance to the $n$-th neighbour where $n$ is usually the minimum sample parameter. HDBSCAN relies on so called mutual reachability distance which is defined between two points $a$ and $b$ as the maximum of the $k$-th core distance of $a$, $k$-th core distance of $b$ or just the distance between $a$ and $b$. In order to split points into clusters a graph of points is constructed with edges connecting nodes weighted by mutual reachability distance. We can split the graph into clusters using standard DBSCAN approach where a threshold value for reachability is chosen. In HDBSCAN we instead construct a minimum

spanning tree using Prim's algorithm. Next, hierarchy of connected components is build by sorting the edges of the minimum spanning tree by distance in increasing order and iterating to create new merged clusters from each new edge, creating a dendogram. Finally, the dendogram is split into clusters by either cutting at a given reachability (equivalent again to DBSCAN) or by iteratively condensing the cluster tree. The objective is to find splitting that leaves a single point out of the cluster. When the procedure starts persistently losing points, instead of splitting into more clusters, a cluster is likely found. All points that get lost consequently belong to a single cluster. This can be controlled by the second parameter in HDBSCAN - minimum cluster size. HDBSCAN can also accept a maximum cluster size as well, but is not necessary.

## 1.3 Reasoning on the choice of clustering algorithms

Previous studies related to conserved water analysis (albeit without subsequent hydrogen orientation analysis) from molecular dynamics simulations used DBSCAN.[1,2] We also considered DBSCAN[3] but opted not to use it after initial testing. It's main disadvantage is that all generated clusters have the same density and clustering parameters influence the results in often unpredictive ways. Furthermore, DBSCAN is very sensitive to two parameters while both HDBSCAN and OPTICS are usually only very sensitive to one. Both OPTICS and HDBSCAN have one main clustering parameter, namely so called minimum sample value, which can also determine minimum cluster size. OPTICS also has a so called $\xi$ parameter which can be used to influence the clustering, but if chosen reasonably it can always remain constant. Newest versions of HDBSCAN has a parameter that can limit maximum cluster size, while OPTICS does not have similar functionality. This functionality can be extremely useful when performing single clustering procedure.

## 1.4 Single clustering procedure

The single clustering procedure involves performing a single clustering pass on the oxygen position data set with the clustering parameters which ensure that size of the clusters obtained has to be between 80% and 120% of the number of snapshots. When the maximum cluster size can't be explicitly controlled in the OPTICS and HDBSCAN algorithms, the clustering often generates clusters of size that is much larger compared to the number of snapshots. This would often manifest by observing oxygen clustering cluster sizes approximately close to the number of snapshots in the trajectory in one part of the system, while very poor results (usually large clusters, much larger than the number of snapshots in trajectory) in another part of the system. This problem can be circumvented by implementing the multi-stage re-clustering procedure. We introduced a re-clustering procedure based on the parameter scan of clustering parameters to combat the issue of poor clustering quality due to the inability to constrain the maximum cluster size.

## 1.5 Benchmarking of clustering algorithms

For benchmarking the dataset of 6H76 (Siap) system was used. The data base of contains 17000 water molecules (which is a reasonable estimate for number of water molecules for several hundred snapshots of water molecules in relatively closed active sites of proteins used in this study). The median of 5 entries which were averaged over 3 runs was taken as a representative execution time for each algorithm. All the calculations were performed on Intel Xeon E5 - 2630 v4 @ 2.2GHz using single thread.

We have performed time scaling analysis to compare relative speed of two clustering algorithms used for oxygen clustering. HDBSCAN's median was 1.1 seconds while OPTICS' median was 55.33 seconds. OPTICS, however often gives higher quality and more reliable clusters then HDBSCAN, but both methods give qualitatively similar results.

We have also compared the run time of HDBSCAN and OPTICS and Kmeans for small data set of 200 orientation inputs for hydrogen orientation clustering. OPTICS run time was

0.311 seconds while HDBSCAN performed in 0.01 seconds. OPTICS is generally slower but we decided the downside of computational time debt is worthwhile the uptake in clustering quality for hydrogen orientation analysis.

We have also benchmarked the single clustering procedure for same data set. The min-samp parameter for single clustering run used was 201 which is equivalent to number of snapshots considered. The HDBSCAN run took 57.16 seconds and consumed 400.94 MB of RAM. The OPTICS run took 77.44 seconds and consumed 401.34 MB of RAM. In multi stage re-clustering approach the run time varies significantly between the systems and between HDBSCAN and OPTICS. In multi stage re-clustering approach many single clustering procedure cycles have to be evaluated but it is extremely difficult to predict how many. Thus the run time for multi stage re-clustering procedure is difficult to estimate since it is often independent of the system size due to how the procedure works, and thus the relative comparison in run time between different systems or even between OPTICS and HDBSCAN is not particularly useful. The RAM usage should be the same as in the case of single clustering procedure because no additional data is ever created or used. The RAM usage will scale with system size (number of water molecules selected for analysis) for both approaches in a very similar fashion.

## 1.6 Detailed explanation of hydrogen orientation analysis

Some operations in the determination of certain water types are identical and will be explained first. In the case of FCW and HCW at least one of the hydrogen orientation clusters must have its size similar to the number of elements in the oxygen cluster to ensure that at least one hydrogen is always pointing in a well defined single direction. This is checked by enforcing that the size of the hydrogen orientation cluster must be between 85% and 115% of the size of the the corresponding oxygen cluster.

The angles between hydrogen orientation clusters can be calculated in multiple ways. However, we must ensure that the value of the angles as well as their variance is sufficiently

tight in order to filter out the unwanted clusters. We opted for the procedure where we calculate the mean orientation in one cluster first and then calculate the mean and the standard deviation between the said orientation and certain other set of orientations usually belonging to a different cluster. This allows us to have a check on the variance of the orientations in the studied set, as well as an average measure of the angle between the two sets of orientations.

Procedures for evaluating all three water types require an input data set of normalised orientations. Orientations are represented by 3 dimensional normalised vectors pointing from the central oxygen to the two corresponding hydrogens. The size of this data set is always twice the size of the number of elements in the studied oxygen cluster. The metric used for the hydrogen orientation clustering is always the standard Euclidian $l^2$ metrics.

### 1.6.1 Fully conserved waters

The schematic representation of the FCW determination is given in Scheme 1. The k-means clustering algorithm from the scikit-learn[4] with 2 clusters is performed on the data set of the hydrogen orientation vectors. If the angle between the centres of two clusters is greater than 120° or the inertia of clusters (the variance) is larger than 0.4 the analysis is stopped and the oxygen cluster under consideration is not considered as a FCW. K-means represents a clustering method where the data is split in predetermined number of clusters by minimising the variance of the data in each cluster. As such k-means is very useful in evaluating the spread (variance) of the clustered data. A FCW must have tight variances in hydrogen orientation and both clusters must the have so-called inertia value less than 0.4 and the angle between the respective cluster centres must be lower than 120°.

If k-means clustering yields satisfactory results, OPTICS clustering is performed with the minimum number of samples equal to 85% of the size of the related oxygen cluster and $\xi = 0.03$ on the same data set. Two largest clusters obtained from OPTICS clustering are checked for size to insure that both orientations are very well defined. The average

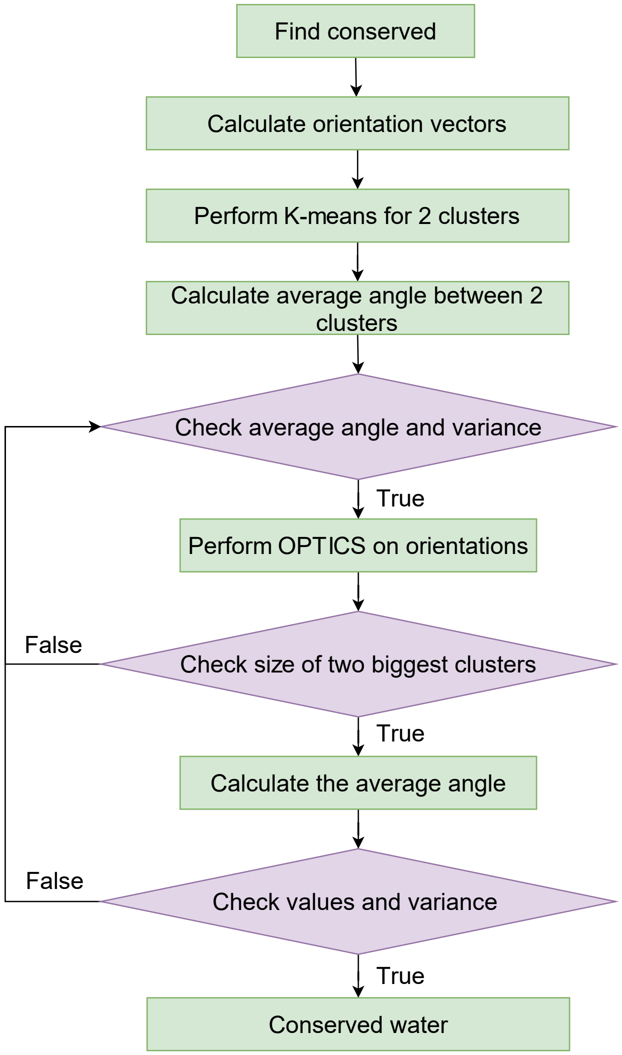


Scheme 1: The algorithm for detecting the FCW molecules.

orientation of both clusters is calculated and the average angle between each of the two average orientations with all orientations belonging to the other cluster is calculated. If the difference between these two angles is smaller than 5° and the standard deviation of both of the means is smaller than 17, the two averaged orientations are accepted as valid orientations of the hydrogen atoms and the water is labelled as a FCW.

### 1.6.2 Half conserved waters

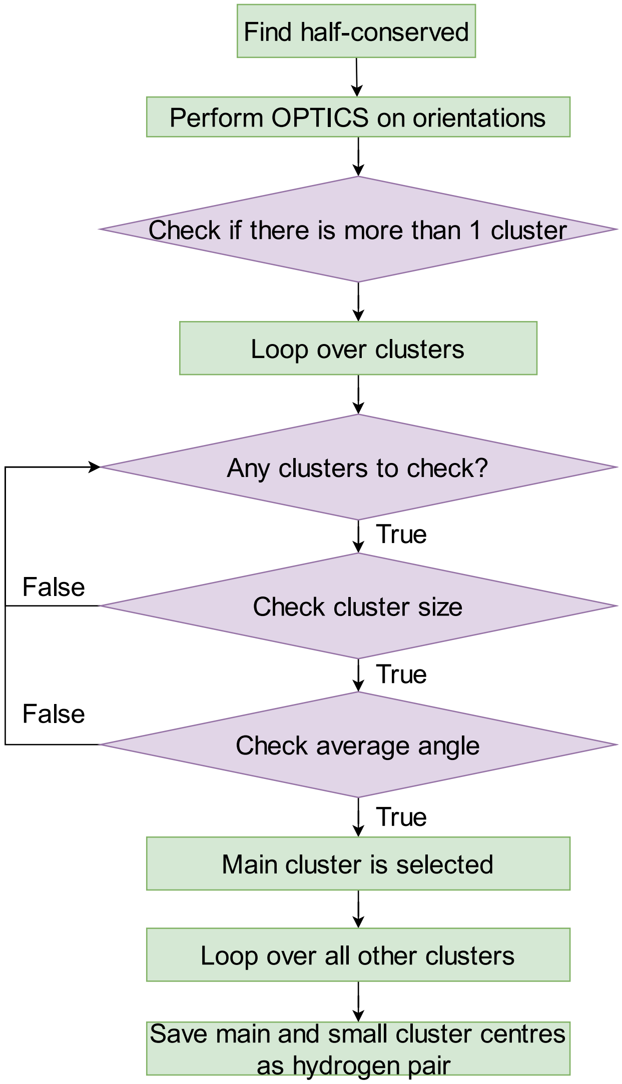


Scheme 2: The algorithm for detecting the HCW molecules.

A check if a given water molecule is HCW begins by performing OPTICS clustering on

hydrogen orientation vectors with a minimum sample size of 35% of the size of the related oxygen cluster (Scheme 2). This enables smaller clusters to be generated by the OPTICS algorithm. First, we check if there is more than a single cluster present, if not the water molecule is discarded. An attempt is then made to find the main hydrogen orientation cluster which is always directed in a single direction. To find this cluster we loop over hydrogen orientation clusters and check their size. Next, we calculate the average angle between the mean orientation of the cluster and all other orientations. If the average angle is between 90° and 120° and the standard deviation is smaller than 17, the cluster is labelled as the main hydrogen orientation cluster, which is always pointing in a single preferred direction.

Next we loop over all the remaining clusters. The average orientations of both the main and remaining clusters are saved for the visualisation. Our code always returns the main cluster as the first hydrogen for visualisation so that the hydrogens with constant orientation (the main hydrogen orientation) from a HCW can be readily visualised as well. We repeat the algorithm presented in Scheme 2 for two different values of $\xi$: 0.05 and 0.01.

### 1.6.3 Weakly conserved waters

The algorithm for the determination of WCW orientations is given in Scheme 3. To find WCW, we perform OPTICS clustering with a minimum sample size of 15% of the size of the relevant oxygen cluster size and with $\xi$ values of 0.05 and 0.001.

First, we attempt to find cluster pairs (doublets) of equal sizes with the water angle in between (see Figure 1 WCW-I). We loop over each unassigned cluster and check if its size lies between 35% and 115% of the size of the oxygen cluster. We allow much smaller sizes in the analysis of WCW to catch the waters with several preferred orientations. Next, we loop over rest of the clusters that haven't been assigned yet. We check if the size of the cluster is equal to the size of the main cluster. We require that the smaller cluster be between 85% and 115% of the size of the main cluster. The average angle between average orientation of the main cluster and all orientations in the new cluster is calculated. If the angle is between

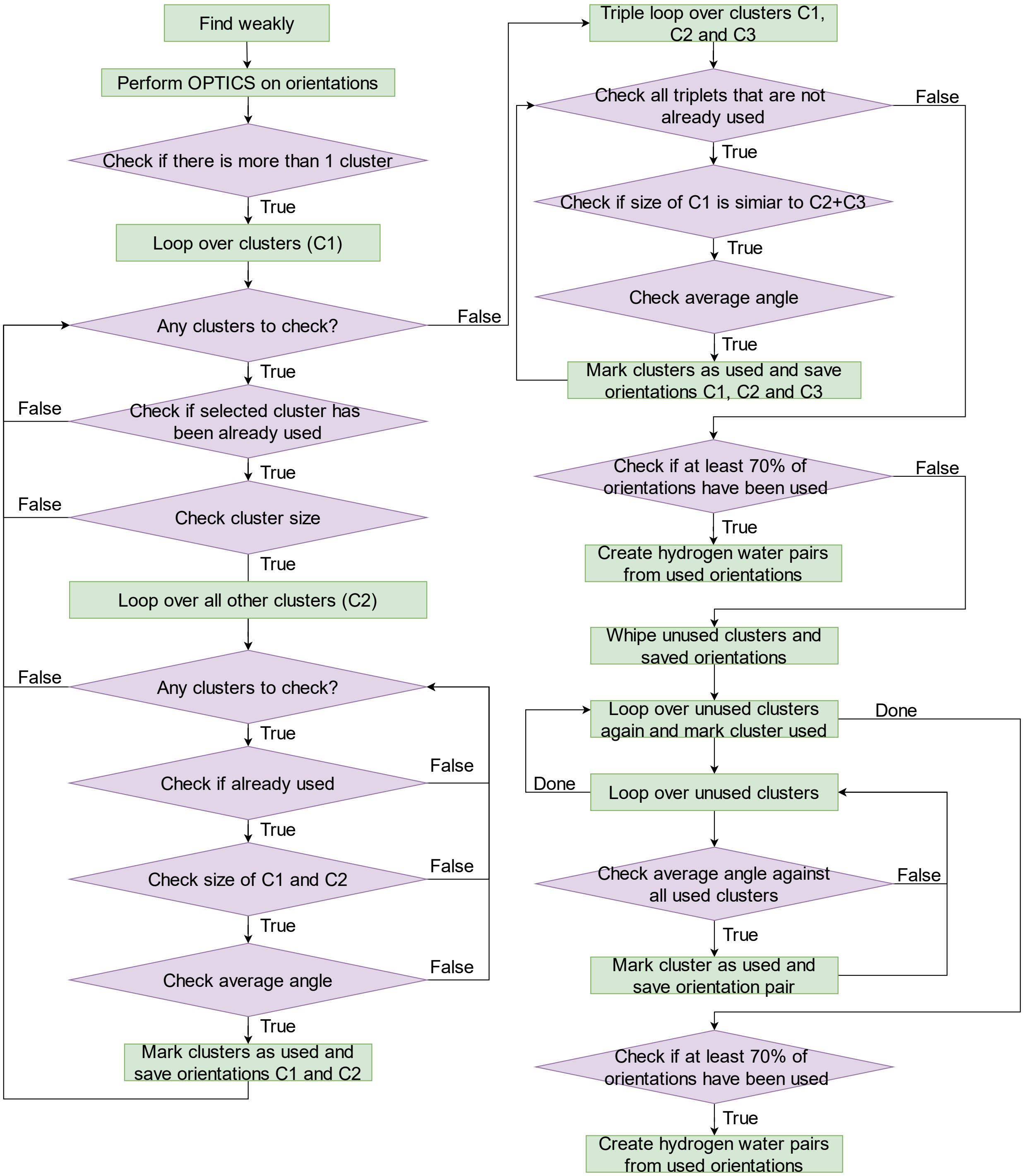


Scheme 3: The algorithm for detecting the WCW molecules.

85° and 115° and the standard deviation is smaller than 17 the average orientations of the two clusters are selected as a pair of possible WCW hydrogen orientations. The two clusters are marked as assigned and the main loop continues looping over the rest of the unassigned clusters.

Triplets of clusters are checked next. It is possible that one hydrogen is oriented in a single direction while the other hydrogen is "jumping" between two possible orientations (see Figure 1 WCW-II). We loop over all unassigned triplets of the hydrogen orientation clusters. The size of the first cluster is compared to the sum of the sizes of the second and third cluster. If the size of the sum of the smaller clusters is between 85% and 115% of the size of the bigger cluster, we check for the angle next. If the average angle between the average orientation of first cluster and all orientations in other two clusters is between 90° and 120° and the standard deviation is below 17 we label the triplet of orientations as assigned. The main triplet loop continues until all unassigned clusters are checked. If the percentage of explained orientations for triplets and pairs is above 70% the water molecule is identified as a WCW and the following part of the algorithm is skipped.

If the percentage of explained triplets and pairs is lower than 70%, we discard the assigned clusters and check for circular multiplets (see Figure 1 WCW-III). For example, a multiplet can occur when two hydrogen orientations are "jumping" between several different acceptors but no hydrogen has a preferred orientation. In this case the size constraints are lifted. We loop over unassigned clusters to select a starting main cluster and a multiplet instance. A secondary loop loops over all the remaining unassigned clusters and checks if the newly selected cluster has a viable water angle between all previously selected clusters in this instance. Once all clusters are exhausted and if the instance contains 3 or more clusters, the clusters with satisfactory angles are marked as assigned. The main loop continues searching for further multiplets of unassigned clusters in a new instance. If the number of orientations explained in this way is greater than 70% we label this water molecule as a WCW.

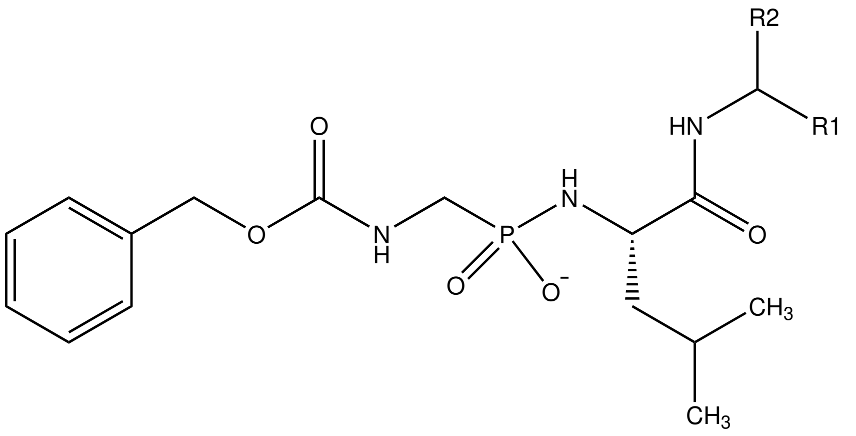

**TLN-1:** R1 = H, R2 = H
**TLN-2:** R1 = $CH_3$, R2 = H
**TLN-3:** R1 = H, R2 = $COO^-$
**TLN-4:** R1 = $CH_3$, R2= $COO^-$

Figure S1: Structural formula of Thermolysin ligand, Cbz-Gly-($PO_2^-$)-L-Leu-$NH_2$-P2' (Cbz = carboxy-benzyl) and its derivatives used in study of Betz et al.[5]

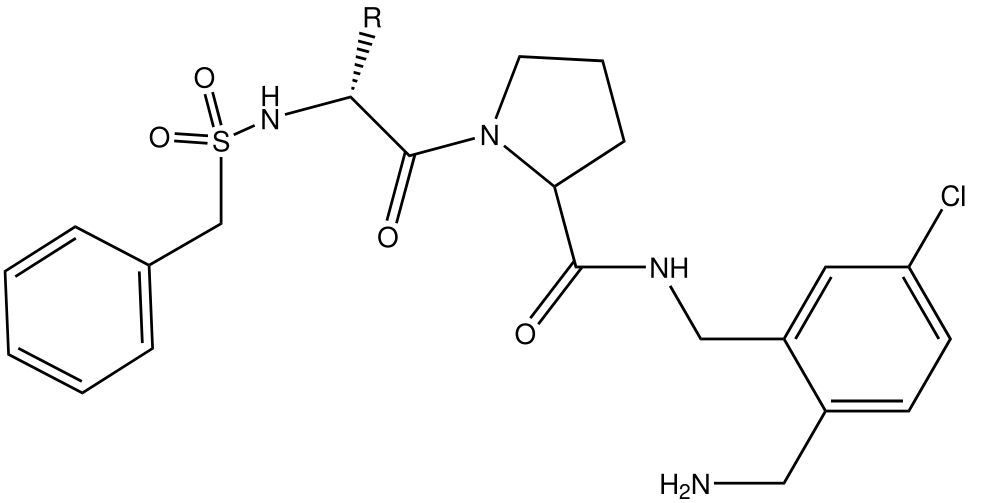

**1:** R = Gly
**2:** R = D-Ala
**3:** R = D-Val
**4:** R = D-Leu
**5:** R = D-Cha

Figure S2: Structural formula of Thrombin ligand, ACB (2-(Aminomethyl)-5-chlorobenzylamide) and its derivatives used in study of Biela et al.[6]

# 2 Water Probability Density Maps

## 2.1 Calculation of water probability density maps from our simulation results

We compute water probability density maps using the same methodology as described in Betz et al.[5] in order to provide a comparison between the results. The water probability maps are computed using VOLMAP plugin through VMD.[7] Volmap type of occupancy was selected with resolution of 0.4 Å. Full radius of oxygen atoms was used and results were averaged over the same frames we use in our clustering analysis.

To generate water probability density maps we select all the water molecules inside a cutoff around the centroid of the protein active site amino acids coordinates. The cutoff used for water density map calculations is the same as the one used in the selection of waters for our clustering analysis. The computed density is exported as .dx file for further visualisation in PyMOL and NGLview.

Our code supports calculation of oxygen density maps using MDAnalysis python package[8,9] version 1.0.0. We use the MDAnalysis' DensityAnalysis method from the density submodule. The main difference is that MDAnalysis only supports the calculation using point particles, thus such results wouldn't give an exact comparison to Betz et al.[5]

### 2.1.1 Comparison of results obtained from clustering with water density probability maps

We compared our clustering results with the water density probability maps calculated from our trajectories for thermolysin, thrombin, and SiaP systems. Excellent agreement for TLN-1 system was observed (see Figure S3 top row, left). For TLN-3 system a discrepancy in W3 is observed while other waters are correctly detected by our clustering scheme. Namely, a substantial water density is detected at W3 position while the clustering approach does not report a conserved water present at this location. To further understand the origin of this

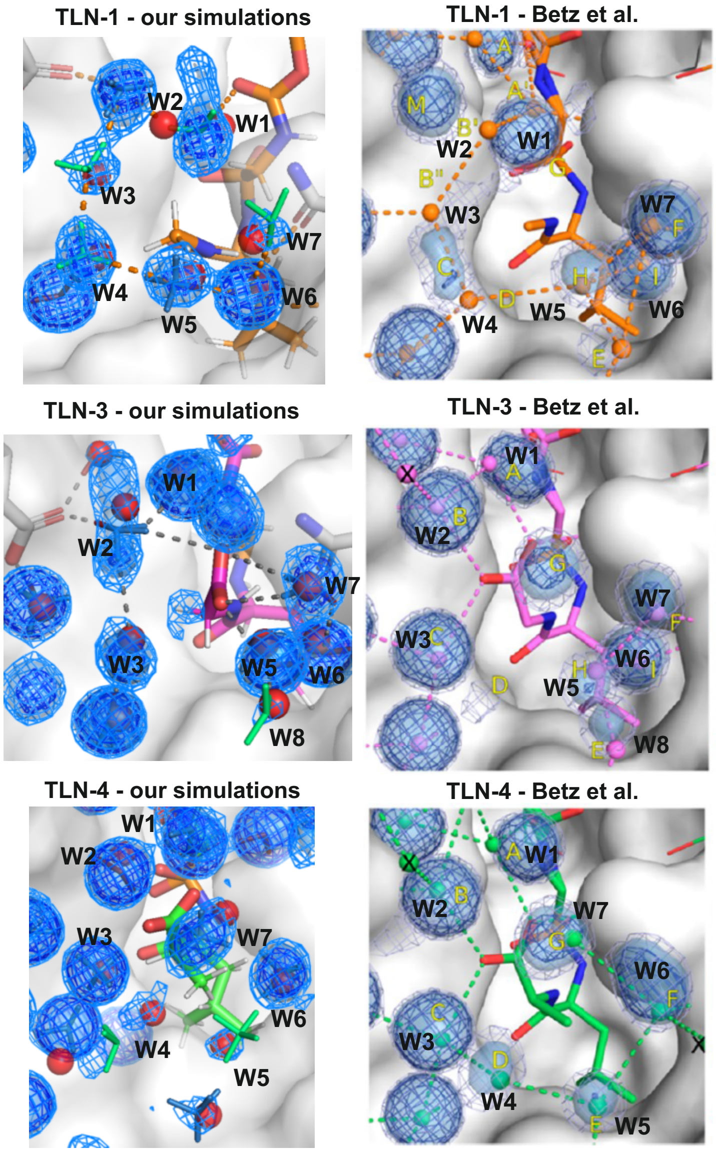


Figure S3: The water probability density maps calculated from our simulations (left) compared to the results of Betz et al. (right).[5] Ligand and water molecules from our clustering analysis (FCW-red, HCW-blue, FCW-green) are shown as large and small sticks, respectively. Red stick cap represents the preferred (main) orientation in HCW. Carbon atoms of ligands 1, 3 and 4 are shown in orange, magenta, and green, respectively, whereas in all complexes blue, red, orange, and yellow are for N, O, P, and S atoms, respectively. The crystal water molecules are represented as red spheres. The light blue mesh indicates regions, where the probability to detect a water molecule along the trajectory, is higher than 52% of the time; the blue transparent surface indicates regions with 60% and the dark blue meshes regions with 68% probability to host a water molecule mimicking the work from Betz et al.

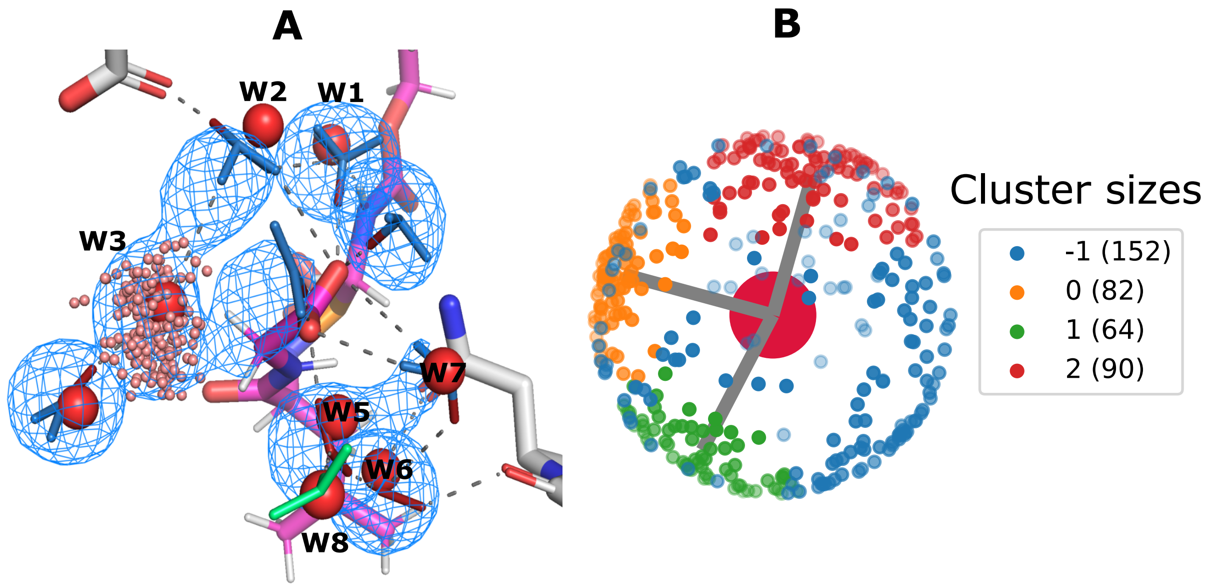


Figure S4: A: The water probability density maps for TLN-3 system. The light blue mesh indicates regions, where the probability to detect a water molecule along the trajectory, is higher than 35% of the time. The small spheres inside W3 density map represent the clustered oxygens detected by oxygen clustering for water W3. B: The hydrogen orientation clustering results for W3 water molecule. Big red sphere represents the oxygen atom. Gray lines represent average cluster orientations. The numbers next to coloured points denote the cluster colours and their sizes obtained from the OPTICS clustering of the hydrogen orientations. The -1 cluster contains data points not assigned to any cluster.

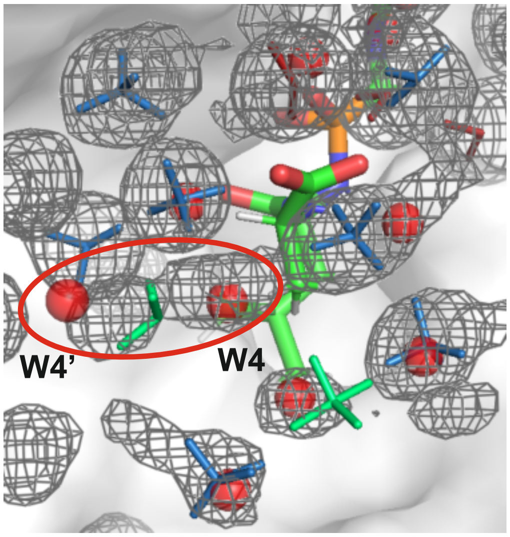


Figure S5: The water probability density map for TLN-4. The gray mesh indicates regions, where the probability to detect a water molecule along the trajectory, is higher than 45% of the time. The red highlighted density represents the density associated with W4 WCW type molecule. Crystallographic waters W4 and W4' are highlighted in the same region of space.

discrepancy we analysed our clustering methodology. We observed that the oxygen clustering correctly detects an oxygen cluster at W3 position (Figure S4 A). However, subsequent analysis of hydrogen orientation clustering showed that the hydrogen orientations were too spread out for the cluster to be considered FCW, and that cluster sizes and their relative angles did not satisfy HCW or WCW (see Figure S4 B). In the TLN-4 case we again observed excellent agreement with the water probability density maps. The W4 water is detected by our clustering approach in between two smaller density peaks (Figure S5). One peak is associated with W4 crystal water while the other one is associated with W4' crystal water. Our clustering analysis classified these two densities into a single water molecule of WCW type. This means that these two crystallographic water molecules (labelled W4 and W4' in Figure S5) are most likely a single water molecule which jumps between the two locations - W4 and W4'. In these situations our clustering approach can still detect a conserved water molecule if the density along this elongated density profile is roughly constant and if the orientations of hydrogens are very similar or very well defined.

In the case of thrombin (Figure S6) and SiaP (Figure S7) systems perfect agreement between probability density water maps and our clustering approach was observed. All dense regions are populated by conserved water molecules obtained from the clustering approach.

## 2.2 Comparison of probability density maps calculated from our trajectories with calculated water probability density maps using simulations from literature

We compared results presented in Betz et al.[5] obtained from calculation of water probability density maps from MD simulations with the probability density maps calculated from our trajectories (see Figure S3). Reader should note slight discrepancies in simulation details, such as slightly different water model (TIP4P vs. TIP4PEW), electrostatics method (PME vs. Ewald) and smaller restraint on protein and ligand atoms (10 kJmol$^{-1}$Å$^{-2}$ vs. 100

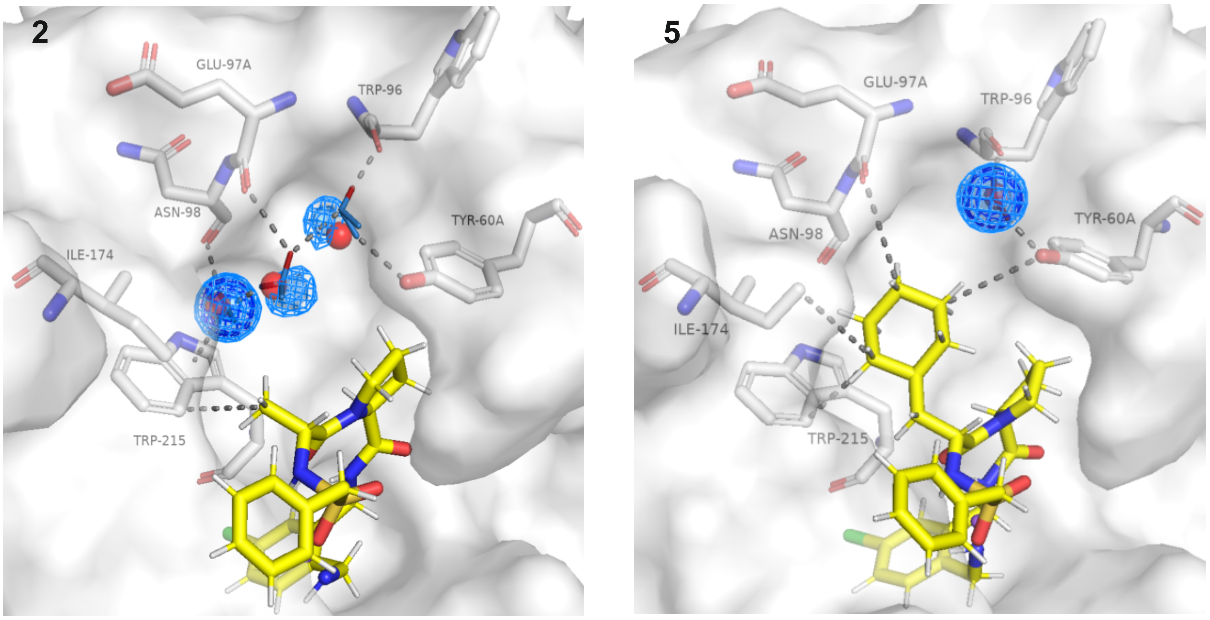


Figure S6: The water probability density maps in thrombin complex **2** and thrombin **5** calculated from our simulations. Water molecules from our clustering analysis are represented by small stick model (FCW-red; HCW-blue). Red stick cap represents the preferred (main) orientation in HCW. Ligands (C yellow, N blue, O red, Cl green) and key amino acids (C gray, O red, N blue) are shown as large sticks. The light blue mesh indicates regions, where the probability to detect a water molecule along the trajectory, is higher than 52% of the time; the blue transparent surface indicates regions with 60% and the dark blue meshes regions with 68% probability to host a water molecule mimicking the work from Betz et al.

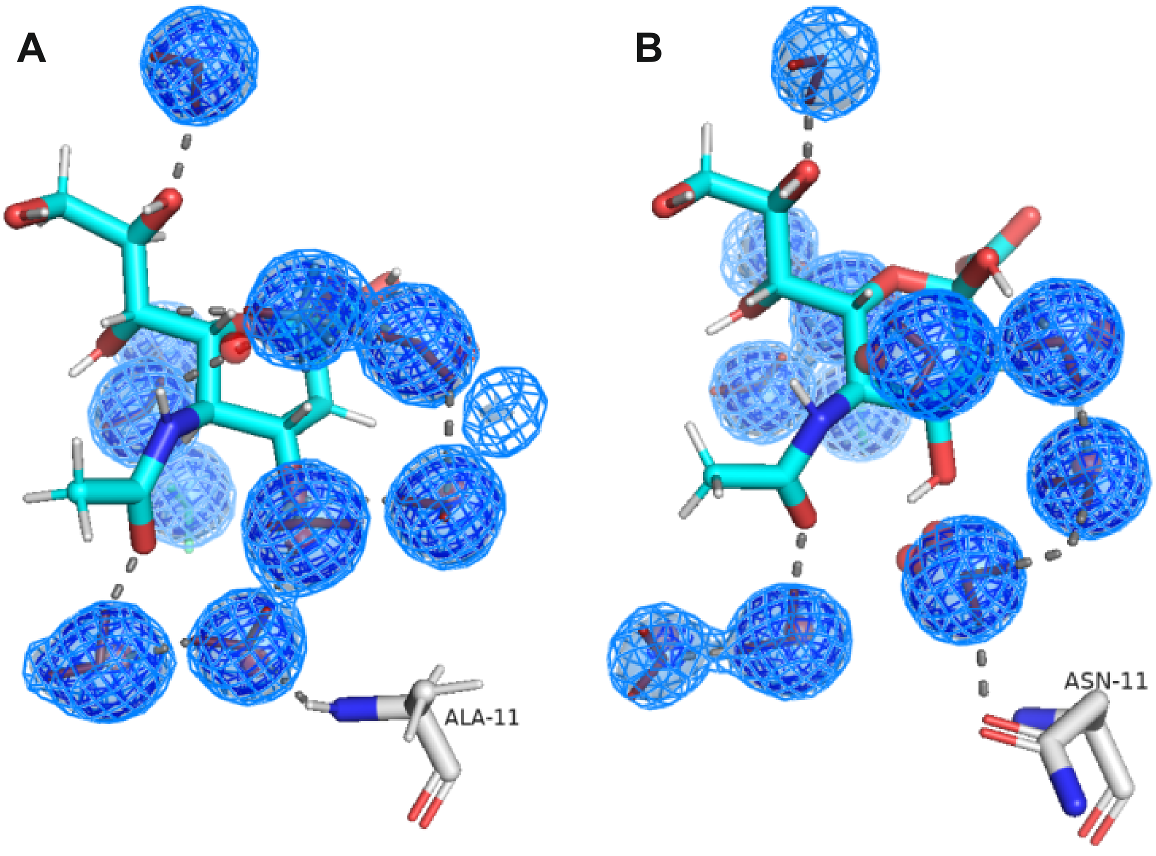


Figure S7: The water probability density maps in SiaP WT(**A**) and A11N mutant (**B**) calculated from our simulations. Water molecules from our clustering analysis are represented by small stick model (FCW-red; HCW-blue, WCW-green). Red stick cap represents the preferred (main) orientation in HCW. Ligands (C cyan, N blue, O red) and key amino acid (Ala or Asn, C gray, O red, N blue) are shown as large sticks. The light blue mesh indicates regions, where the probability to detect a water molecule along the trajectory, is higher than 52% of the time; the blue transparent surface indicates regions with 60% and the dark blue meshes regions with 68% probability to host a water molecule mimicking the work from Betz et al.

kJmol$^{-1}$Å$^{-2}$) in our case.

The only discrepancy in the case of TLN-1 system represents the elongated crystal water site C in Figure S3 top right), which is reproduced as two separate probability density meshes in our case (top left). The TLN-3 system shows almost perfect match between the water density maps from our simulations and simulations of Betz et al (Figure S3 middle left and right respectfully). The only very slight discrepancy is detected in W2 water which in our case shows very elongated water density. For TLN-4 system very good agreement is observed again (Figure S3 bottom). The only discrepancy is in the water density of W4 which is very elongated in our case - the W4 and W4' are merged into a single elongated water density, while the simulations of Betz et al. show two distinct well defined water densities for these two waters.

# References


(1) Jukič, M.; Konc, J.; Janežič, D.; Bren, U. ProBiS H2O MD Approach for Identification of Conserved Water Sites in Protein Structures for Drug Design. *ACS Med. Chem. Lett.* **2020**, *11*, 877–882.

(2) Jukič, M.; Konc, J.; Gobec, S.; Janežič, D. Identification of Conserved Water Sites in Protein Structures for Drug Design. *J. Chem. Inf. Model.* **2017**, *57*, 3094–3103.

(3) Ester, M.; Kriegel, H.-P.; Sander, J.; Xu, X. A Density-Based Algorithm for Discovering Clusters in Large Spatial Databases with Noise. Proceedings of the Second International Conference on Knowledge Discovery and Data Mining. 1996; p 226–231.

(4) Pedregosa, F. et al. Scikit-Learn: Machine Learning in Python. *J. Mach. Learn. Res.* **2011**, *12*, 2825–2830.

(5) Betz, M.; Wulsdorf, T.; Krimmer, S. G.; Klebe, G. Impact of Surface Water Layers on Protein-Ligand Binding: How Well Are Experimental Data Reproduced by Molecular

Dynamics Simulations in a Thermolysin Test Case? *J. Chem. Inf. Model.* **2016**, *56*, 223–233.

(6) Biela, A.; Sielaff, F.; Terwesten, F.; Heine, A.; Steinmetzer, T.; Klebe, G. Ligand Binding Stepwise Disrupts Water Network in Thrombin: Enthalpic and Entropic Changes Reveal Classical Hydrophobic Effect. *J. Med. Chem.* **2012**, *55*, 6094–6110.

(7) Humphrey, W.; Dalke, A.; Schulten, K. VMD: Visual Molecular Dynamics. *Journal of molecular graphics* **1996**, *14*, 27–28.

(8) Michaud-Agrawal, N.; Denning, E. J.; Woolf, T. B.; Beckstein, O. MDAnalysis: A Toolkit for the Analysis of Molecular Dynamics Simulations. *J. Comput. Chem.* **2011**, *32*, 2319–2327.

(9) Gowers, R. J.; Linke, M.; Barnoud, J.; E Reddy, T. J.; Melo, M. N.; Seyler, S. L.; Domá nski, J.; Dotson, D. L.; Buchoux, S.; Kenney, I. M.; Beckstein, O. MDAnalysis: A Python Package for the Rapid Analysis of Molecular Dynamics Simulations. *PROC. OF THE 15th PYTHON IN SCIENCE CONF* **2016**,
